# An experimental study of acoustic bird repellents for reducing bird encroachment in pear orchards

**DOI:** 10.3389/fpls.2024.1365275

**Published:** 2024-09-09

**Authors:** Qing Chen, Jingjing Xie, Qiang Yu, Can Liu, Wenqin Ding, Xiaogang Li, Hongping Zhou

**Affiliations:** ^1^ Co-Innovation Center of Efficient Processing and Utilization of Forest Resources, Nanjing Forestry University, Nanjing, China; ^2^ College of Mechanical and Electronic Engineering, Nanjing Forestry University, Nanjing, China; ^3^ Nanjing Institute of Agricultural Mechanization, Ministry of Agriculture and Rural Affairs, Jingsu, China; ^4^ Institute of Pomology, Jiangsu Key Laboratory for Horticultural Crop Genetic Improvement, Jiangsu Academy of Agricultural Sciences, Nanjing, Jiangsu, China

**Keywords:** crop protection, bird infestation, sonic bird repellent, pest management, adaptability studies

## Abstract

Bird invasion will reduce the yield of high-value crops, which threatens the healthy development of agricultural economy. Sonic bird repellent has the advantages of large range, no time and geographical restrictions, and low cost, which has attracted people’s attention in the field of agriculture. At present, there are few studies on the application of sonic bird repellents in pear orchards to minimize economic losses and prolong the adaptive capacity of birds. In this paper, a sound wave bird repellent system based on computer vision is designed, which combines deep learning target recognition technology to accurately identify birds and drive them away. The neural network model that can recognize birds is first trained and deployed to the server. Live video is captured by an installed webcam, and the sonic bird repellent is powered by an ESP-8266 relay switch. In a pear orchard, two experimental areas were divided into two experimental areas to test the designed sonic bird repellent device, and the number of bad fruits pecked by birds was used as an indicator to evaluate the bird repelling effect. The results showed that the pear pecked fruit rate was 6.03% in the pear orchard area that used the acoustic bird repeller based on computer recognition, 7.29% in the pear orchard area of the control group that used the acoustic bird repeller with continuous operation, and 13.07% in the pear orchard area that did not use any bird repellent device. While acoustic bird repellers based on computer vision can be more effective at repelling birds, they can be used in combination with methods such as fruit bags to reduce the economic damage caused by birds.

## 1 Introduction

**Figure 1:**
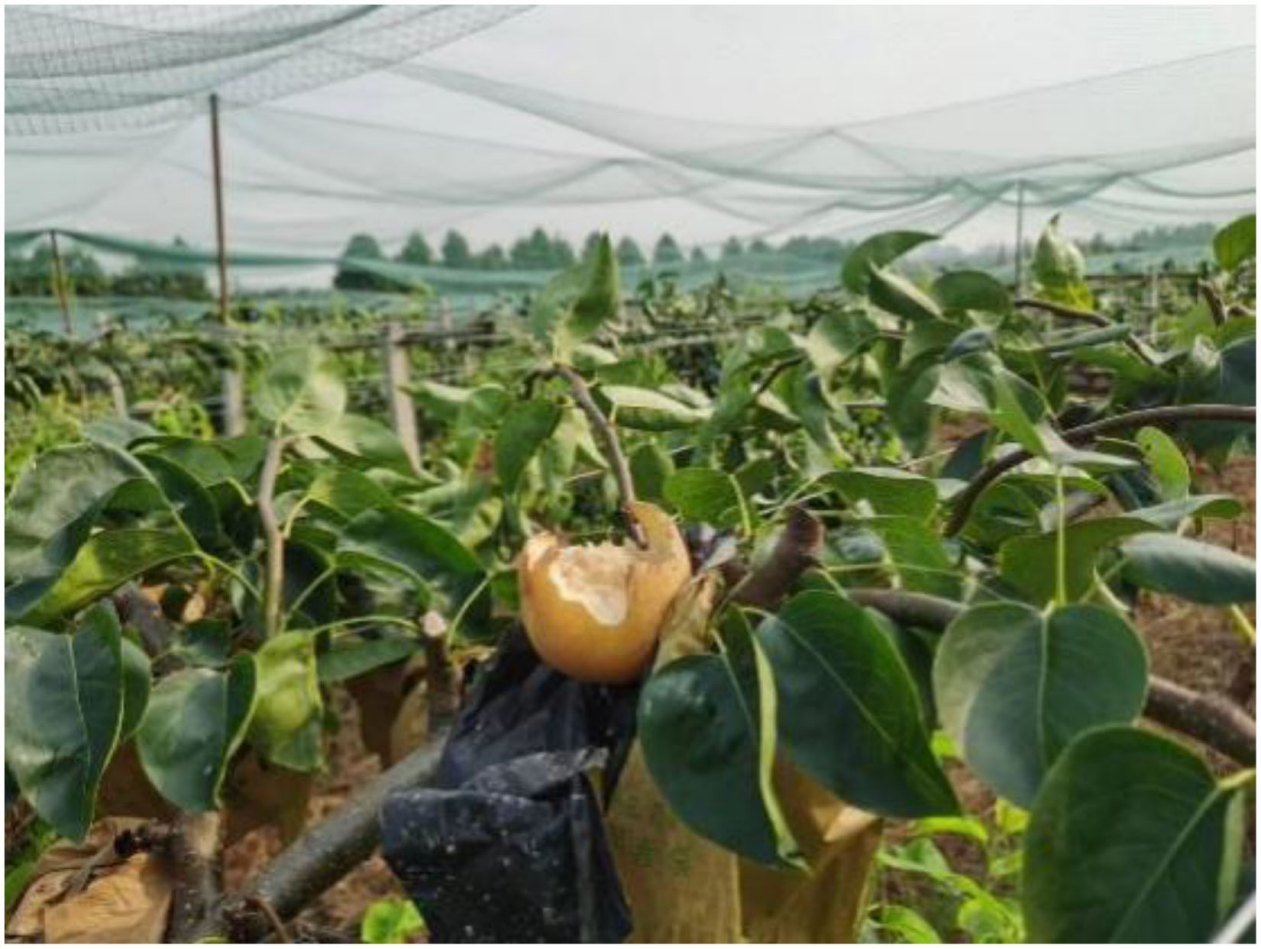
Pear pecked by a bird.

Most orchards are located in suburban areas, which usually have lakes and other sources of irrigation water nearby, and these environments create favorable conditions for bird foraging and roosting. Although birds also play a role in spreading pollen during the fruiting period, avian pollinators require more energy than bees. The bright colors of the petals and the fresh scent of the ripe fruit during this period attract large numbers of birds to peck at the fruit (Coimbra et al., 2020; Sausse and Lévy, 2021). A very conservative estimate for the total loss of commercial crops to pest bird damage in Australia was around $300M AUD annually in 2007 (Wang, 2021). In North Dakota, blackbirds (several spp.) caused annual losses of US$1.3 million in corn (*Zea mays*) production (Peisley et al., 2015). In California alone, more than $49 million was lost to wine grape due to birds, and more than $78 million was lost to five Washington crops (Blueberry, Wine grape, Honeycrisp apple, Sweet cherry, and Tart cherry) (Anderson et al., 2013). Agricultural bird damage is also a serious problem in China. According to a survey conducted by the Beijing Fruit Industry Association, the annual loss of fruit production in Beijing due to bird damage can be up to 80 million kilograms (Lu and Yu, 2011). According to the survey data of other major fruit growing areas, the annual loss of orchards due to bird pests can reach 15%, and even 30% in some areas (He, 2023). In addition to causing a decrease in fruit yield, fruits pecked by birds leave scars, resulting in lower fruit quality, which is very detrimental to the quality development of Chinese fruits (**Figure 1**). Birds pecking pears will also induce other diseases. The fruit is pecked by birds and the juice flows out, which combines with yeast, acetic acid bacteria and various microorganisms in the beaks of birds, eventually causing the fruit to rot and deteriorate. The sour smell emitted attracts vinegar flies to feed and lay eggs (Kross et al., 2012), and spreads and spreads various diseases among fruit trees.

Bird pests cause huge losses to economies worldwide, but the effective control of bird pests remains a daunting task. In the past, pest birds, defined as birds causing harm or damage to crops, were dealt with by shooting or poison trapping (Cheke and Sidatt, 2019). However, this practice tends to cause ecological damage (Cowan et al., 2015). With the development and implementation of and sustainable concepts, a range of ‘green’ bird repellent technologies have emerged. The current common method of bird protection in orchards is to bag the fruit or pull bird-proof nets over the whole orchard, which is not only labor-intensive but also generates a lot of management costs (**Supplementary Figure S1**). Some rare bird species are also often entangled in bird-proof nets. Protecting the crop without harming the birds is one of the factors to be considered in the bird repelling process (Firake et al., 2016).

The removal of birds from orchards can be done by sight, sound and habitat. Scarecrows, reflective tape, etc. will visually scare birds. These traditional methods of bird repellent are effective in the early days, but over time birds soon adapt and they become less effective (Ahmad et al., 2018). The bird-repelling effect of automatic lasers becomes different with different bird species. In tests, lasers were less effective in repelling pigeons and more effective in repelling crows (Blackwell et al., 2002). Some studies have also shown that noise from propane cannons and pyrotechnic bursts can repel birds, but after frequent use birds can develop some adaptations that reduce the repelling effect. In addition, noise pollution from propane cannons has become a significant source of conflict between farmers and their neighbors, leading to vandalism, boycotts, threats of lawsuits, and legislative bans on the use of propane cannons in agriculture (Brown and Brown, 2021). Shooting is also a common method of controlling bird damage, with shotguns shooting birds and also eliciting fear in them (Linz et al., 2015). However, this practice is not desirable and most birds are protected animals in China. Some chemicals have also been designed to repel birds. Chemical bird repellents are irritants and can cause physical aversion in birds. Seedlings and seeds of certain plants can be treated with methyl anthraquinone and anthraquinone to reduce pecking by birds (Werner et al., 2010). Chemical bird repellents can reduce bird damage, but fruits may be left with pesticide residues, which not only pose a threat to human and environmental health, but also lead to contamination of drinking water and food and a reduction in biodiversity (Zaffaroni and Bevacqua, 2022). Reducing the use of chemicals protects human health and the environment as well as biodiversity (Plénet et al., 2023), so chemical bird repellents are not common. There are other ways to keep birds out of the orchard, one of which is to alter the landscape features around the orchard to reduce nesting and roosting. But the landscape around the orchard is difficult for us to change without government approval. Another idea to change the habitat of birds is to attract some predators to breed in the area, which can be used to limit the number of birds with biological characteristics and reduce the damage to fruits to some extent (Lindell et al., 2018). However, the introduction of species is more complicated, the input cost is higher, and the effect of bird repellent is not stable, so this method of bird repellent is not commonly used in life.

In general, aural and visual means of bird repelling are more suitable for bird repelling due to their safety and environmental friendliness. Sonic bird repellents have the advantages of large coverage area, low cost and safety control, making them one of the most widely used tools to repel birds. The range of hearing of the human ear is 20-20kHz. Sound waves beyond 20kHz are called ultrasound, which are inaudible to humans and do not cause any discomfort to the human body. However, most birds are sensitive to sound waves in the 25k~35kHz range. Sound waves in this frequency range can interfere with the nervous and physiological systems of birds, thus causing discomfort, and can therefore be used to repel birds (Jenni-Eiermann et al., 2014). But with the complex environment of orchards, the light is easily blocked by leaves when birds are traveling between trees, which affects the effectiveness of laser bird repellents. Compared to laser bird repellents, sound can easily penetrate branches and leaves, therefore, it is feasible to use ultrasound as a bird repellent tool, which has been confirmed by many scholars' studies (Griffiths, 1988; Matsyura et al., 2016). In addition, compared to other traditional bird repellent methods, ultrasonic bird repellers are harmless to humans and other animals while protecting crops from bird damage. More importantly, it does not cause environmental pollution, which is in line with the concept of sustainable development.. Until now, in order to prevent birds from adapting prematurely, such bird repellents, which stimulate birds aurally, have evolved from a single ultrasonic mode to a combination of multiple voice modalities. Berge et al. (2007) developed an audio circuit that plays four different distress calls of crows for use in reducing bird damage in almond groves. Wang et al. (2019) designed a drone that constantly plays predator calls, incorporating bird psychology to effectively control bird damage in the vineyard.

Based on observations of bird behavior, most studies have shown that complex sound waves are more effective at repelling birds than a single audio (Ogochukwu et al., 2012). However, there has been little research into whether the duration of operation of acoustic bird repellents affects the effectiveness of bird repellency. Therefore, an acoustic bird repellent triggered by computer vision was designed and tested in a pear orchard in a separate field. However, the birds that appear in orchards are very good at hiding themselves in complex environmental backgrounds, and their bodies are very small, and it is very difficult to accurately identify such targets. Taleki combined a CNN-based detector with a full convolutional network and hyperpixel-based semantic segmentation via a vector machine to achieve high-performance detection of small objects in large images, reaching high detection performance in bird recognition (Takeki et al., 2016). Lee studied a detection method using deep learning combined with background difference method to identify birds, which first uses background difference method to extract the approximate outline of bird area, and then input it into the convolutional neural network of deep learning for recognition, which can effectively eliminate background interference, but the actual recognition effect is not ideal (Lee et al., 2019). Zhou added the attention mechanism inside the convolutional network model, using the attention mechanism to enhance the local bird image features to improve the classification performance, and based on this feature developed a WeChat applet-based waterfowl image recognition application, which is simple and convenient to use, but the accuracy of its recognition decreases drastically when the number of bird images is insufficient (Zhou et al., 2022). Chen developed an automatic wild bird repelling system, which utilizes mask R-CNN for wild bird recognition and detection and uses a laser to repel wild birds after capturing their locations (Chen et al., 2024). Yu et al. improved the YOLOv3-tiny model to make the algorithm faster by reducing the number of convolution kernels, in addition to employing binocular stereo vision and coordinate transformation to obtain the distance and angle of the bird with respect to the laser, which subsequently controls the gimbal and the laser to perform bird repelling (Yu et al., 2023). Xie adds a sound detection module to the traditional image recognition-based bird repeller, converts bird songs into pairs of mellows as input features, and uses the improved MobiletNet-RNN for bird recognition, to study a new type of bird repeller that is mainly based on sound detection and supplemented by image recognition. However, sound recognition relies on the processing of raw audio by audio technology, otherwise it is susceptible to environmental noise interference, which affects the accuracy of recognition (Xie, 2021). Based on LoRa technology for IoT communication and YOLO target detection algorithm, Xu uses ultrasonic pulses to stimulate the nervous system of birds, combined with high-frequency flashing lights and simulated sounds of natural enemies to realize the repulsion of birds (Xu et al., 2021). In this paper, a real-time ultrasonic bird repellent device is proposed by combining the current state of the art of bird repellent technology. We have studied the recognition system and hardware of the bird repeller separately: 1) Evaluate three recognition algorithms, YOLO, SSD, and RetinaNet, in terms of recognition rate, recognition accuracy, and portability. 2) Aiming at the shortcomings of the YOLOv5 algorithm, we embed a CBAM module in Neck to enhance the ability of the whole model to focus on important features and improve the recognition effect of bird targets in complex environments. 3) Reconstruct the feature fusion structure of YOLOv5 to be more suitable for small-scale bird target recognition. 4) Conduct a field partition experiment in a pear orchard to evaluate the adaptive effect of the designed acoustic bird repeller by the number of birds pecking pears. The experiment showed that the acoustic bird repeller designed in this paper reduced 53.86% of bird-damaged bad fruits than the blank group during the test period in this pear orchard, which provides a reference for promoting intelligent bird repellent in agroforestry.

## 2 Materials and methods

**Figure 2:**
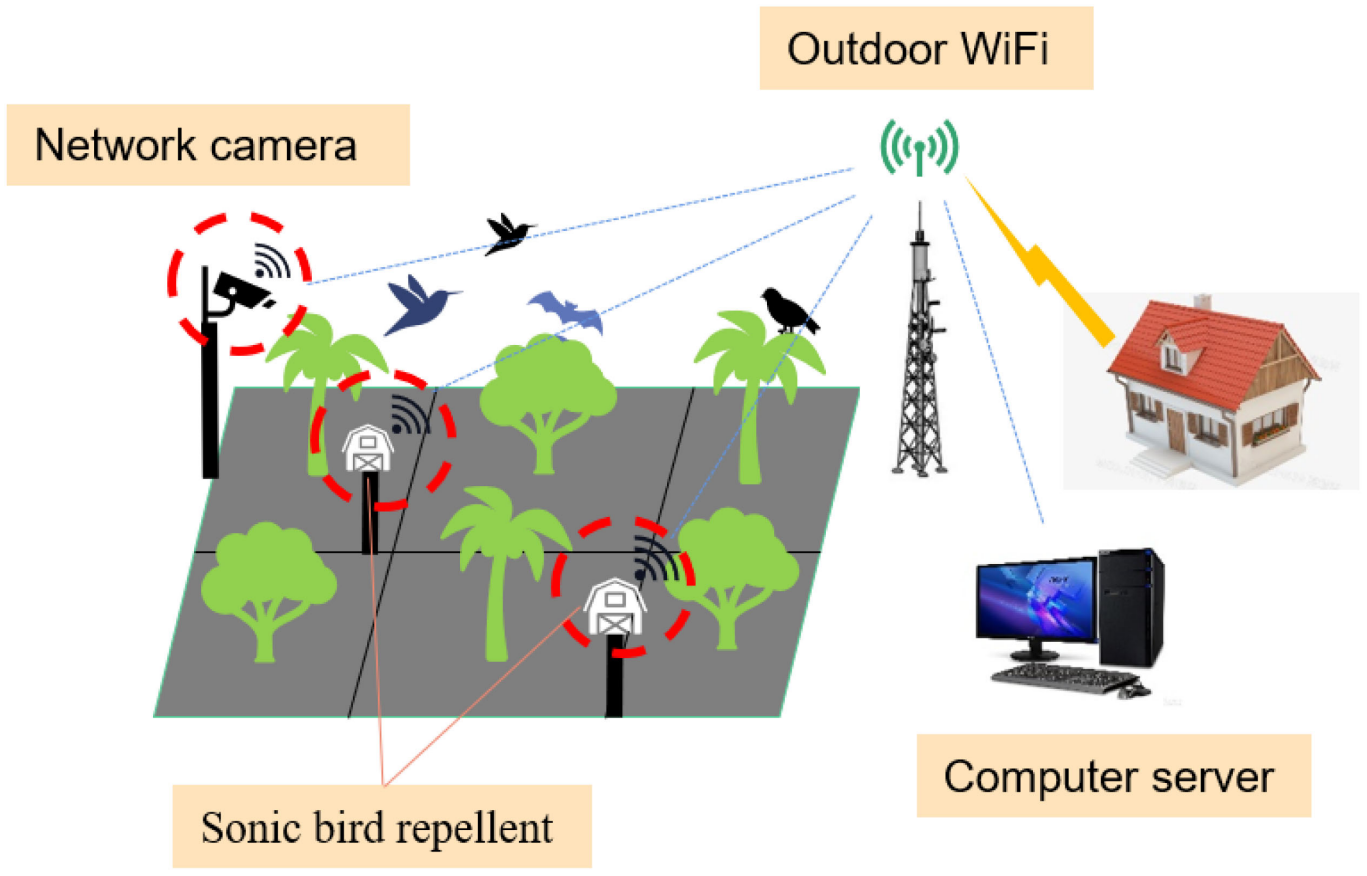
Bird repellent system architecture.

The architecture of the intelligent bird repellent system proposed in this paper consists of a webcam that was installed at an appropriate location in the pear orchard to monitor the presence of flocks of birds above the orchard (**Figure 2**). A sonic bird repeller was installed in the center of the pear orchard to repel pest birds. The computer server is an essential part of deploying the deep learning target detector. Last but not least is an outdoor router that provides the communication medium for the aforementioned devices in the pear orchard.

### 2.1 The repellent device

#### 2.1.1 Digital audio players

The Arduino UNO microcontroller can read digital audio files that were saved on a micro SD card that was used for bird recapture (Iniyaa et al., 2021), which contains many different types of audio, such as cries and explosions of the bird’s natural predators. The Arduino UNO development board is the most commonly used version of the Arduino family, with a programmable core, data processing, data storage, timer/timer, serial and bus communication, pulse width modulated signals, analogue multiplexer, A/D converter, etc. It can be programmed to control the I/O at will for electrical signal control, acquisition and electronic communication, with a rich open source library to greatly improve development efficiency (Siahaan et al., 2017).

#### 2.1.2 Ultrasound player

Device supplied with high frequency square wave signal by IC555 (Agustin et al., 2021). The IC555 is an integrated chip for analogue and digital signals. It is a commonly used timer that outputs a square wave through the charging and discharging of a capacitor, and the rate at which the capacitor is charged and discharged determines the frequency of the square wave it outputs, with an input voltage of 4.5 to 16V.

#### 2.1.3 Power amplifiers

The LM386 is a mono low-voltage amplifier with a supply voltage of 4~18V and a driving load range of 4~32Ω. It has the advantages of low power consumption, adjustable voltage gain, large supply voltage range and low total harmonic distortion, which is widely used in radios and hearing aids. The internal principle of the LM386 is a three-stage amplifier circuit. The first stage is a differential amplifier circuit, which uses a mirror current source as the active load of the differential amplifier circuit, so that the gain of the single-ended output circuit is approximately equal to the gain of the double-ended output capacitor. The second stage is the main gain stage, which is a common-source amplifier circuit employing a constant-current source as the active load to increase the amplification. The third stage is a quasi-complementary symmetrical power amplifier with the introduction of deep negative voltage feedback and a stable voltage gain throughout the circuit. In practice, a resistor R and a capacitor C are connected between the Pin8 and Pin1 pins so that the voltage amplification can be increased to 200dB gain by adjusting the resistance and capacitance values.

#### 2.1.4 Wi-Fi modules

The designed bird repellent needs to establish communication with the computer server to receive signals back from the server after identifying the target. Currently, the main wireless technologies that can realize the Internet of Things are Bluetooth, Zigbee, Wi-Fi, GPRS, etc. **Table 1** summarizes the advantages and disadvantages of the above four commonly used wireless communication technologies. Bluetooth is low-power and inexpensive, but has low data transfer rates. Bird recognition in the experiment is detected in real time and bird repellers are turned on when the presence of a bird is detected, which requires high image transmission speeds. However, the transmission speed of Zigbee is too low, only up to 250kbps, which cannot meet the purpose of real-time monitoring. In addition, compared with Zigbee, Wi-Fi technology is relatively mature and has a wider range of application scenarios. GPRS has no restriction on distance and has a long transmission distance, but it is more expensive. In order to meet the experimental requirements, we eventually considered using Wi-Fi as the data communication method between the various parts of the bird repellent system after comprehensive consideration. The ESP8266 Wi-Fi module can be connected to a Wi-Fi network via the TCP/IP protocol, operates at 2.4 GHz, and sends and receives data using a serial communication UART (Zhang and Tao, 2020; Liaw and Li, 2021). This development only needs to use the Wi-Fi communication module to send and receive signals and does not need to use it as the main control board. Therefore, the inexpensive and practical ESP8266-01 module was chosen to be used in conjunction with the relay that accompanies the module to control the on/off of the audio circuit (Balakrishna et al., 2021).

**Table 1:** Comparison of wireless communication technologies.

Technology	Advantages	Disadvantages	Distance/m	Applications
Bluetooth	Cheap, low power	low transmission speed	10-200	Smart home, wearable devices
Zigbee	Low power consumption, low latency, large network capacity	Weak ability to penetrate walls and low transmission speed	20-350	Industrial, automotive, smart home
Wi-Fi	easy to use, low cost	poor stability, high power consumption	20-350	Smart home, open public areas
GPRS	Fast speed, long transmission distance	High cost	Unlimited	Industrial, Medical

#### 2.1.5 Power supply module

**Figure 3:**
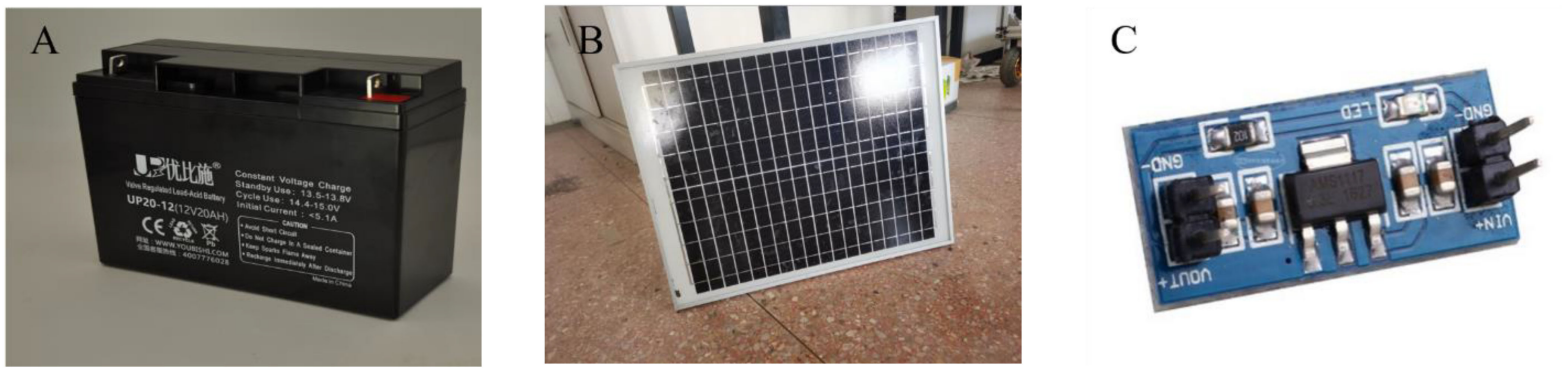
Power supply module for acoustic bird repellent. **(A)** 12V lead-acid batteries for powering equipment. **(B)** Photovoltaic panels allow equipment to hold more electricity during the day. **(C)** AMS1117 voltage regulator module provides stable on-board power for development boards.

Bird repellents are generally used in wilderness areas far from human habitation, where power supply is difficult and long working hours are required. Therefore, the design considers the use of a 12V lead-acid battery as the driving power source for the bird repellent circuit board and the use of a photovoltaic panel to provide continuity for the battery. The relay operates at 5V and the battery at 12V, so a step-down module is required. The module used is the AMS1117 power regulator, which provides on-chip overload and overheat protection and is inexpensive at only $0.28 (**Figure 3**).

#### 2.1.6 Web cameras

For outdoor use, the selected camera (P40A2-WT, Dahua Technology Corporation Limited, Zhejiang city, China) supports IP67 level of waterproofing. The camera, whose parameters are shown in **Supplementary Table S1**, can be connected to Wi-Fi to transmit digital video streams from the pear orchard to a target monitor on a computer server, which is very easy to use without wiring. We fixed the webcam on a bracket about four meters high and installed it around the pear orchard to monitor the target area.

#### 2.1.7 Outdoor routers

The outdoor router used has a wireless transmission rate of 1200 Mbps, which is sufficient for high-speed transmission of video and has a coverage radius of 300 meters, which is sufficient to cover the test area. The router provides the network between the network cameras, acoustic bird repellers, computer servers and other devices and was located in an open area near the pear orchard.

### 2.2 Object detect

#### 2.2.1 Datasets

**Figure 4:**
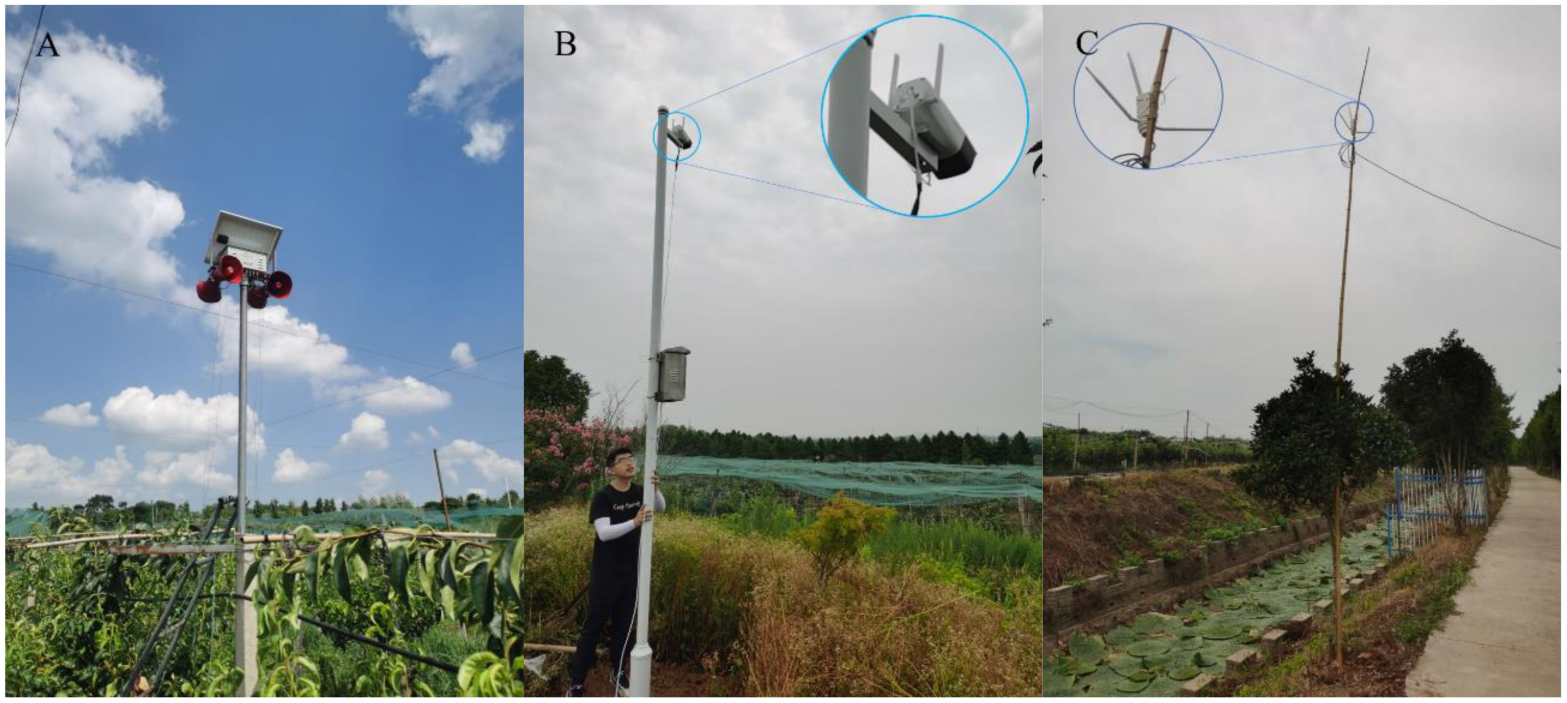
Equipment erected in the pear orchard. **(A)** This paper designs an acoustic bird repeller, which is placed in a pear orchard. **(B)** Network cameras were installed on the edge of the pear orchard to monitor the trial area above. **(C)** The outdoor router was placed near the Pear Garden and the network signal covered the trial area.

**Figure 5:**
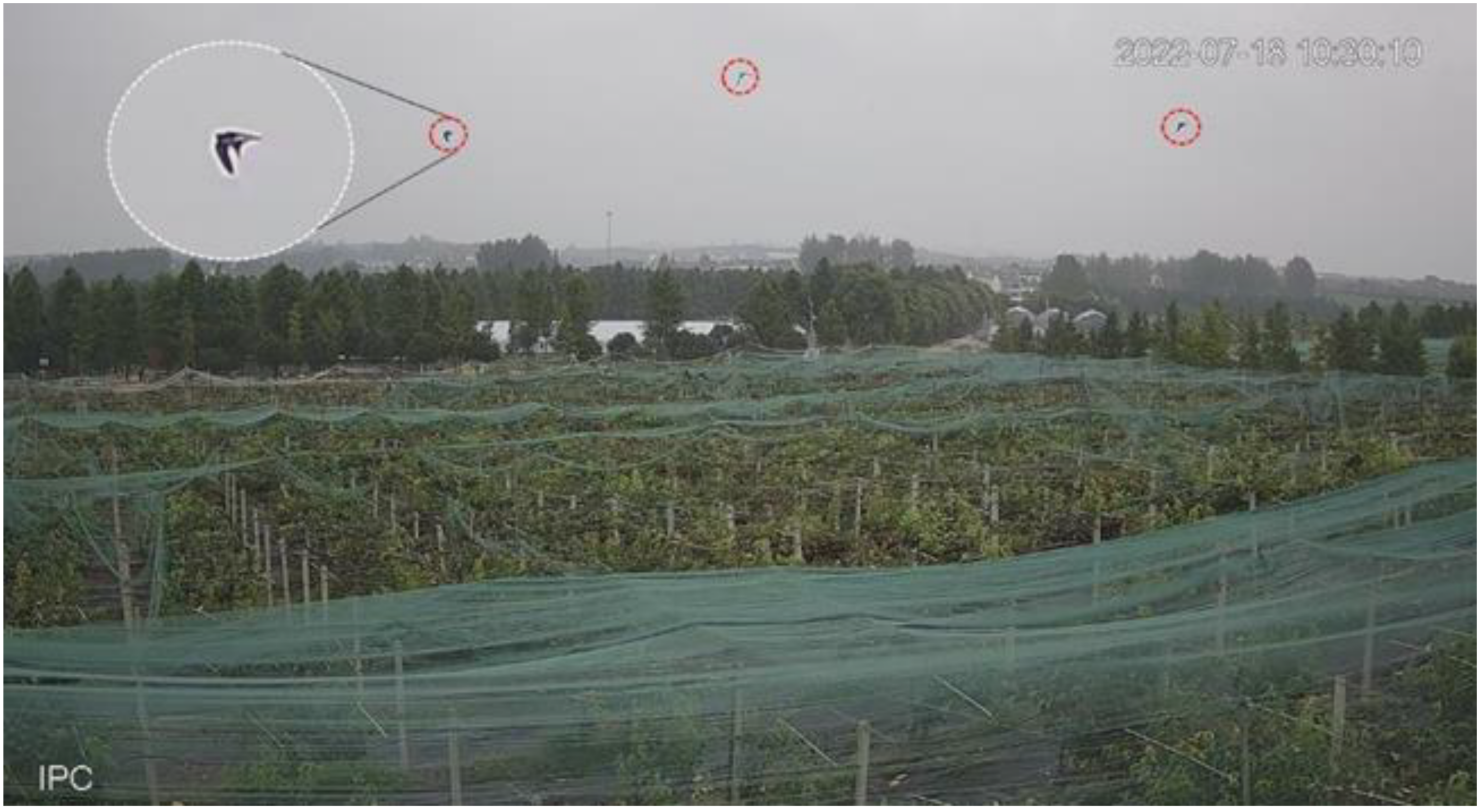
The birds that appear in orchards have very small targets.

In the complex natural environment of an orchard, the flight posture of birds is constantly changing, with many external factors affecting the construction of the bird dataset, such as lighting factors, weather factors, background complexity, motion blur, and other complex factors (Yoshihashi et al., 2015). Therefore, the images in the dataset should be as clear and natural as possible, which is a prerequisite to ensure effective training of deep learning models (Anjana et al., 2021). A number of open source bird datasets also provide a large number of bird images for training. CUB200-2011, a benchmark image dataset released by Caltech for the study of fine-grained classification and recognition of birds, contains 200 subclasses of birds, with very clear physical features of the birds (Verstraeten et al., 2010). For studying bird recognition in orchards in natural environments, images collected in the field were very helpful in improving the recognition rate of the model. We used a video camera which was set up in the pear orchard as shown above (**Figure 4**) to capture video images of birds in distant, close up and complex backgrounds at different times of the day and under different lighting conditions. The video captured by the camera has a resolution of 1920 x 1080 pixels and was saved locally in mp4 format. We wrote a script using opencv-python that intercepted frames from the video, preserved the video frame images and the birds in the frames, and saved them in JPEG format (**Figure 5**). The above acquired images along with some bird photos from the open source dataset constitute the final dataset for training the deep learning target detector. We named this set of 10,000 bird images Bird-Mix. We labeled these images with labelImg software and placed the saved xml file in the Annotations folder of the YOLOv5 target finder and the images in the images folder.

#### 2.2.2 YOLOv5 model

YOLOv5 is a deep learning target detection algorithm that combines speed and accuracy, consisting of Input, Backbone, Neck and Prediction (Zhu et al., 2021). Input consists of 3 main components: mosaic data enhancement, adaptive anchor frame calculation and adaptive image scaling. Backbone consists of Focus, Cross Stage Partial Network (CSP) and Spatial Pyramid Pooling (SPP). The Focus structure slices the image to obtain a down-sampled feature map with 2 times more information; the CSP structure is designed to solve the problem of excessive computation in inference from the perspective of network structure design; the SPP layer increases the perceptual field and enhances the non-linear representation of the network by maximum pooling after three convolutions of the feature layer.

The Neck part of YOLOv5 uses a combination of Feature Pyramid Network (FPN) and Path Aggregation Network (PAN), where the FPN gradually propagates deep semantic information from top to bottom to shallow layers, delivering strong semantic features and smoothing different scales. Then, PAN passes contour and localization information top-down in the shallow network, which enhances the feature extraction capability of the feature pyramid structure. Taking an input image of 640×640 size as an example, after five times of down sampling, the output feature map size is 20×20. The YOLOv5 algorithm presets 9 anchor points of different sizes through K-means clustering, and every 3 anchor points are a group, to predict the targets of three scales: large, medium, and small.

Based on the bird dataset Bird-Mix produced in the previous section, the training and test sets were divided in a 9:1 ratio, and a total of 800 training batches were trained, each with a sample size of 24. The SGD function was used for training to optimize the parameters, with an initial learning rate of 0.01, a momentum coefficient of 0.937 and a weight decay coefficient of 0.0005. The training took a total of 28h, with a final average detection accuracy of 89.9%.

**Figure 6:**
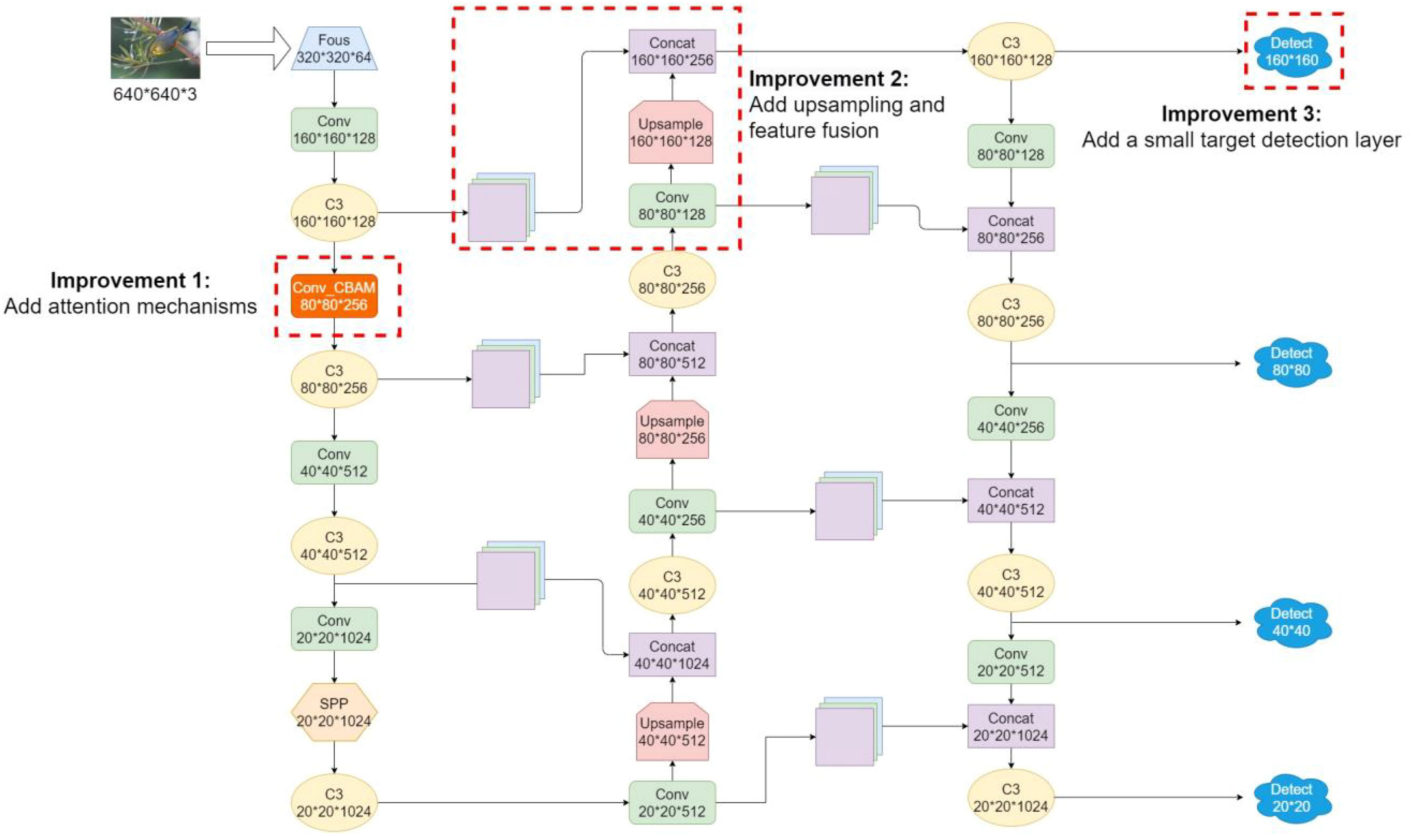
Modified YOLOv5 network structure.

YOLOv5 acts as a convolutional neural network to obtain the feature information of targets in an image by convolution, but the resolution of the original image is greatly reduced after multiple convolutions. A 640×640 size image is reduced in size by 32 times after 5 times of downsampling, while a 32×32 size target in the original image is only 1×1 size after convolution, and targets smaller than 32×32 will lose all the information. In this case in **Figure 5**, the lack of feature extraction during the training process brings great difficulties to bird recognition. In the Neck network of the original YOLOv5, the feature maps obtained by 8× down sampling, 16× down sampling and 32× down sampling of the original image are stitched and fused multiple times, and finally three feature maps of different sizes are output for detecting targets of different sizes. On the basis of YOLOv5s structure, the identification of small target birds is optimized. On the basis of the original 80×80 small-target detection feature map, a detection layer with a size of 160×160 was added to improve the sensitivity of the model for small-target bird recognition (Guo et al., 2022). At the same time, by simulating the biological visual processing, the weights learned by the neural network are dynamically weighted to strengthen some important information and suppress some unimportant information (Liu et al., 2020). A hybrid attention mechanism called convolutional Block Attention Module was introduced in computer vision to improve the algorithmic model’s ability to extract features from targets in specific regions of the frame and to weaken the interference of the complex environmental background of the orchard on the recognition of bird targets. The essence of the attention mechanism is to weight the convolved features according to the feature relevance, and the newly added attention mechanism can improve the ability of the network model to extract important features and suppress unnecessary features (Chen et al., 2020). Meanwhile, the structure of the feature fusion network was improved by adding up-sampling to convey deep semantic features from top-down, and the path fusion network conveyed the location information of the target from bottom-up, which can improve the sensitivity of the YOLOv5 model to small targets and the ability to capture targets in complex environments. Finally, a small target detection layer was added to avoid losing the feature information of small targets and to effectively learn the feature information of shallow and deep feature maps. **Figure 6** shows the modified YOLOv5 network structure with several improvements to the original YOLOv5.

In order to demonstrate the superiority of the improved YOLOv5 algorithm in bird small target recognition, it is compared with SSD, YOLOv3, YOLOv4 and the unimproved YOLOv5 algorithm. The experimental configuration environment is shown in **Table 2**.

**Table 2:** Hardware and software environment parameters of the experiment.

Configuration Name	parameters of a version
display card	NVIDIA GeForce RTX 2080Ti
processors	i9-9900K
random access memory (RAM)	64GB
operating system	Windows 10 Professional
Deep Learning Framework	Pytorch
CUDA	11.1
Opencv	4.5.5.62
Python version	3.8

The evaluation metrics of the experimental results included precision (P), recall (R), average precision (mAP@0.5), and frames per second (FPS), and the experimental results were shown in **Table 3**. At IOU=0.5, a higher mAP value indicated a better recognition effect; a higher FPS indicated a shorter prediction time for a single image.

**Table 3:** Comparison of experimental results.

Detection models Evaluation metrics	P	R	mAP@0.5/%	FPS(frames/second)	File size (MB)
SSD-VGG16	0.766	0.624	65.9	23.4	91.6
YOLOv3	0.912	0.838	88.9	52.63	117
YOLOv4	0.864	0.802	87.2	32.98	244
YOLOv5s	0.932	0.825	89.9	62.5	13.7
Improved YOLOv5	0.911	*0.848	*92	62.3	15.0

* and bold values indicate that the improved algorithm in this paper has better results.

It is easy to see from **Table 3** that while the SSD algorithm has the fastest detection speed, it also has the lowest accuracy. Among a series of YOLO algorithms, YOLOv4 is relatively poor in terms of both mAP and detection speed. YOLOv3 improves the mAP by 1.7% and the frame rate by 19.65 when compared to YOLOv4, while YOLOv5 improves the mAP by 2.7% and the frame rate by 29.52 when compared to YOLOv4. The average accuracy is improved by 2.1% when the improved YOLOv5 algorithm compared with the YOLOv5s. Although the FPS is reduced by 0.2 due to the addition of the feature fusion layer and the CBAM module, the real-time monitoring requirements for the frame rate are generally greater than 25 frames. The frame rate of the improved YOLOv5 algorithm in this paper is 62.3, which is far more than the standard requirement.

**Figure 7:**
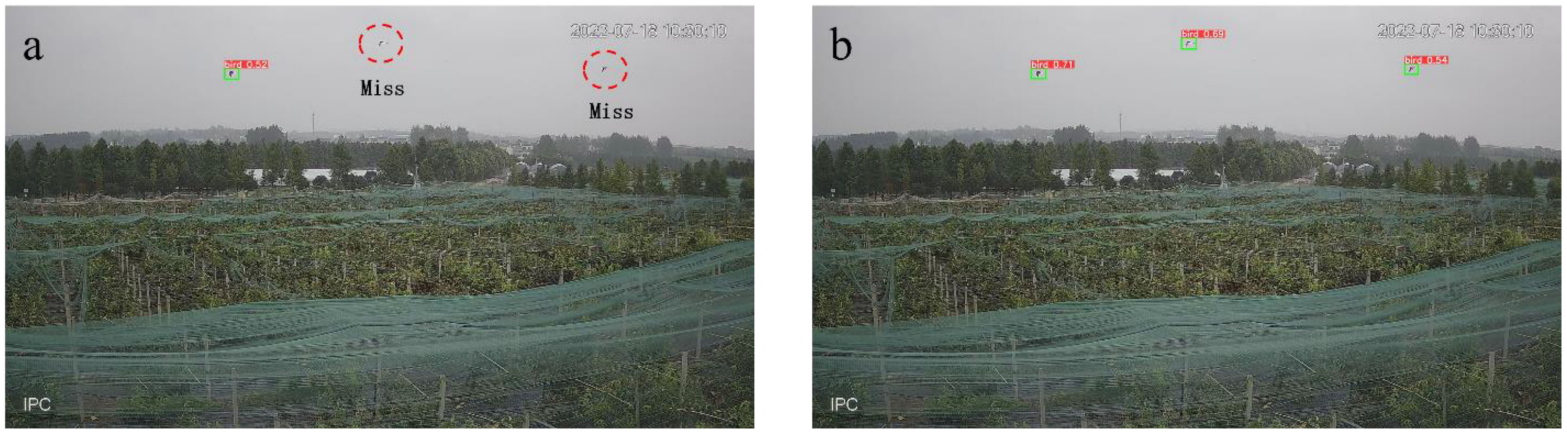
Comparison of recognition effects before and after the improvement of the YOLOv5 model. **(A)** Two birds were not recognized before the improvement. **(B)** After the improvement, all three birds were recognized.

The average accuracy of the improved YOLOv5 training is 92%, which is 2.1% higher than before the improvement. The final trained weights file was used to test the improved YOLOv5 target detection algorithm for recognizing bird targets in the pear orchard captured by the webcam, and the results can be seen in **Figure 7**, which shows that the bird targets in the pear orchard can be accurately recognized even though they are very small.

In this paper, by reconstructing the feature fusion network structure of the YOLOv5 algorithm model, the feature information of different scales and layers can be effectively integrated to solve the problem of losing the feature information of small-target birds caused by excessive convolution operation. In addition, the introduction of the attention mechanism can fully extract bird feature information and enhance the robustness of the model in recognizing bird targets. The above two improvements can retain and utilize the subtle features of small targets more effectively, so as to enhance the robustness and generalization ability of the YOLOv5 model in recognizing bird small targets in the complex environment of orchards.

#### 2.2.3 Ablation experiment

The ablation comparison experiment is to verify the optimization effect of each improved module on the Bird-mix dataset with the original YOLOv5s target detection network as the baseline, and the experimental results are shown in **Table 4** below.

**Table 4:** Ablation experiments on Bird-mix.

Dataset enhancement	Add CBAM	Multiscale feature fusion	mAP@0.5/%
			89.9
√			90.9
√	√		90.5
√		√	91.2
√	√	√	**92**

The bold shows that the improved algorithm in this paper has better results.

By analyzing **Table 4**, it can be concluded that the dataset enhancement has improved the generalization of the model, resulting in a 1% improvement in mAP. A slight decrease in mAP was observed after the addition of the two CBAM attention modules. This phenomenon may be due to the fact that the two sets of CBAM modules replaced the original Conv module, thus affecting the effectiveness of feature extraction by the backbone network. In the third set of experiments, after increasing the number of network layers and fusing shallow and deep features several times, the mAP reached 91.2%. Increasing the number of network layers strengthened the feature extraction capability and ensured that the information extracted from the shallow layer could be transferred to the deep feature map through multi-scale feature fusion, which improved the accuracy of bird recognition, especially for small and medium-sized targets. In the last set of experiments, the CBAM module weights the input features from both spatial and channel dimensions, which helps the network to pay more attention to the important features in the input image and suppresses the background interference. Meanwhile, by adding a detection layer, it compensates for the decrease in feature extraction ability caused by replacing the Conv module with the CBAM module in the two groups, and finally improves the mAP to 92%.

### 2.3 Experimental design

**Figure 8:**
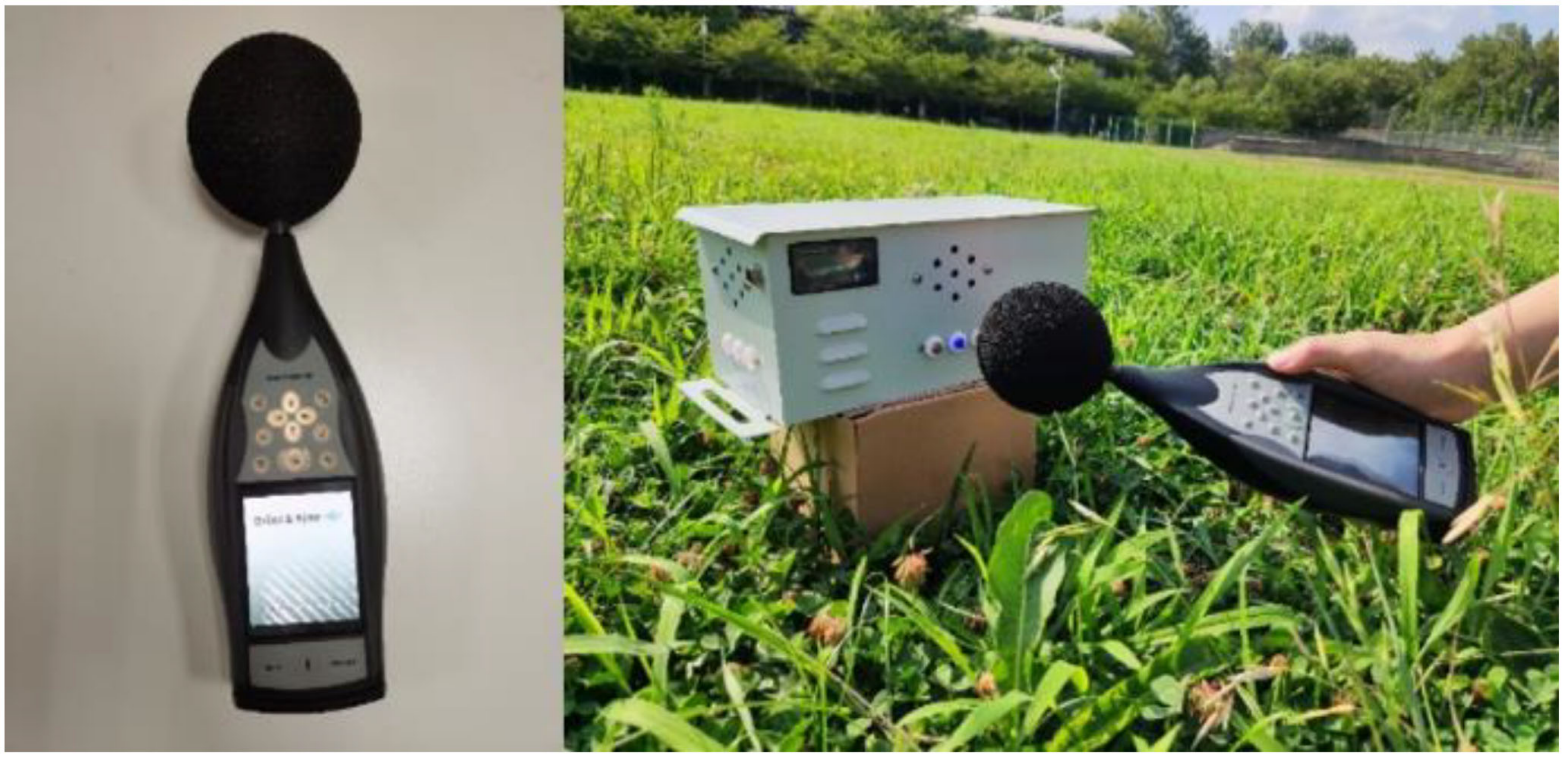
Bruel & Kjaer Sound Level Meters.

Prior to the test, the sound pressure level of the designed acoustic bird repellent was measured to facilitate the development of a specific research program. The instrument used is a Bruel & Kjaer brand acoustic measuring instrument (**Figure 8**). A sound level meter, also known as a noise meter, is a noise measuring instrument. Sound level meters convert acoustic signals into electrical signals, simulating the sensitivity of the human ear to different frequencies of sound waves. A-weighted sound level is the most meaningful and widely used for describing human ear hearing relative to the real acoustic frequency response, so the frequency response data of bird repellents are recorded in this paper with A-weighted sound pressure level.

As can be seen from **Table 5**, the designed bird repellent has a sound pressure level of 113.4dB (A), which can meet the normal bird repelling operation requirements. The environmental noise at the time of measurement was 49.2 dB(A), which can be taken as the environmental noise sound pressure level, and the sound pressure level gradually decreased as the sound level meter was further away from the bird repeller (Fitzwater, 1970). At a sound level meter distance of 16m from the bird repellent, the acoustic bird repellent sound pressure level was 53dB (A), so it can be judged that the bird repellent was placed at a distance of 24m at a comparable sound pressure level to when it was not placed.

**Table 5:** Sound pressure level of acoustic bird repellent in relation to sound level meter distance.

Distance/m	Sound pressure level/dB(A)
0	113.4
8	79.2
16	53
Environmental sound	49.2

**Figure 9:**
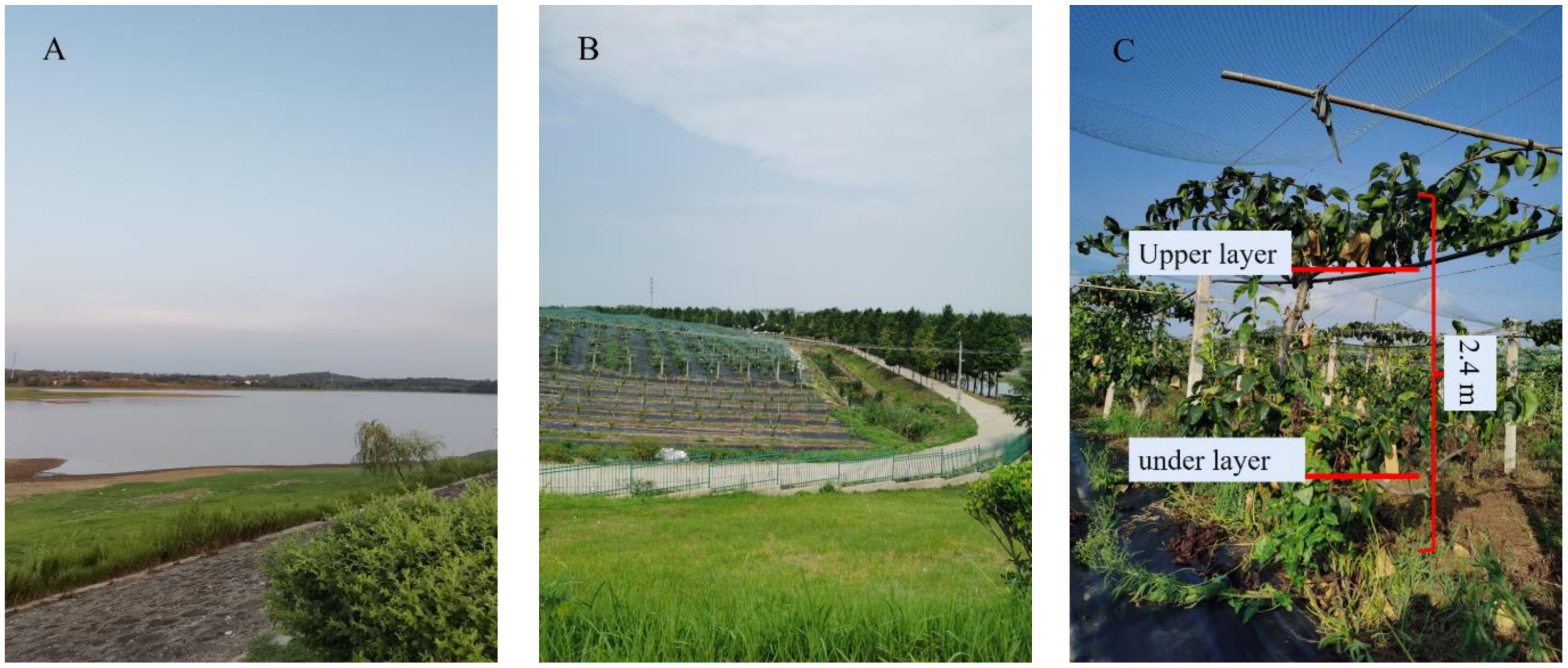
**(A)** The orchard is bordered by the “Oriental Red” reservoir, from which water is drawn for the irrigation of the orchard’s fruit trees. **(B)** The orchard is surrounded by hills and has a high degree of forest cover, making it a very suitable environment for birds. **(C)** View inside the Pear Garden.

The experimental site is an orchard in Liuhe district, Nanjing city, Jiangsu province, China, at the Lvhang Agricultural Plantation (N: 32°21′12.09″, E: 119°0′57.16″). The fruit grown in the experimental area (**Figure 9B**) is pear, with a harder skin than other berry fruits, which starts to ripen in July. Pears ripe to attract birds around to peck, mainly crows, magpies, tits, pheasants and other medium-sized birds. The orchard’ s current bird-proofing strategy is to bury steel pipes near the fruit trees, which are covered with large bird-proof nets made of wire. The owner of the pear orchard also indicated that this method of bird control has a huge investment cost, and the material and labor cost of the bird-proof netting is also invested more each year. Due to the attraction of the fruit and the suitable surrounding environment, the orchard attracts a large number of birds to peck at it during the fruiting period (Matsyura, 2018) and is heavily infested with birds.

**Figure 10:**
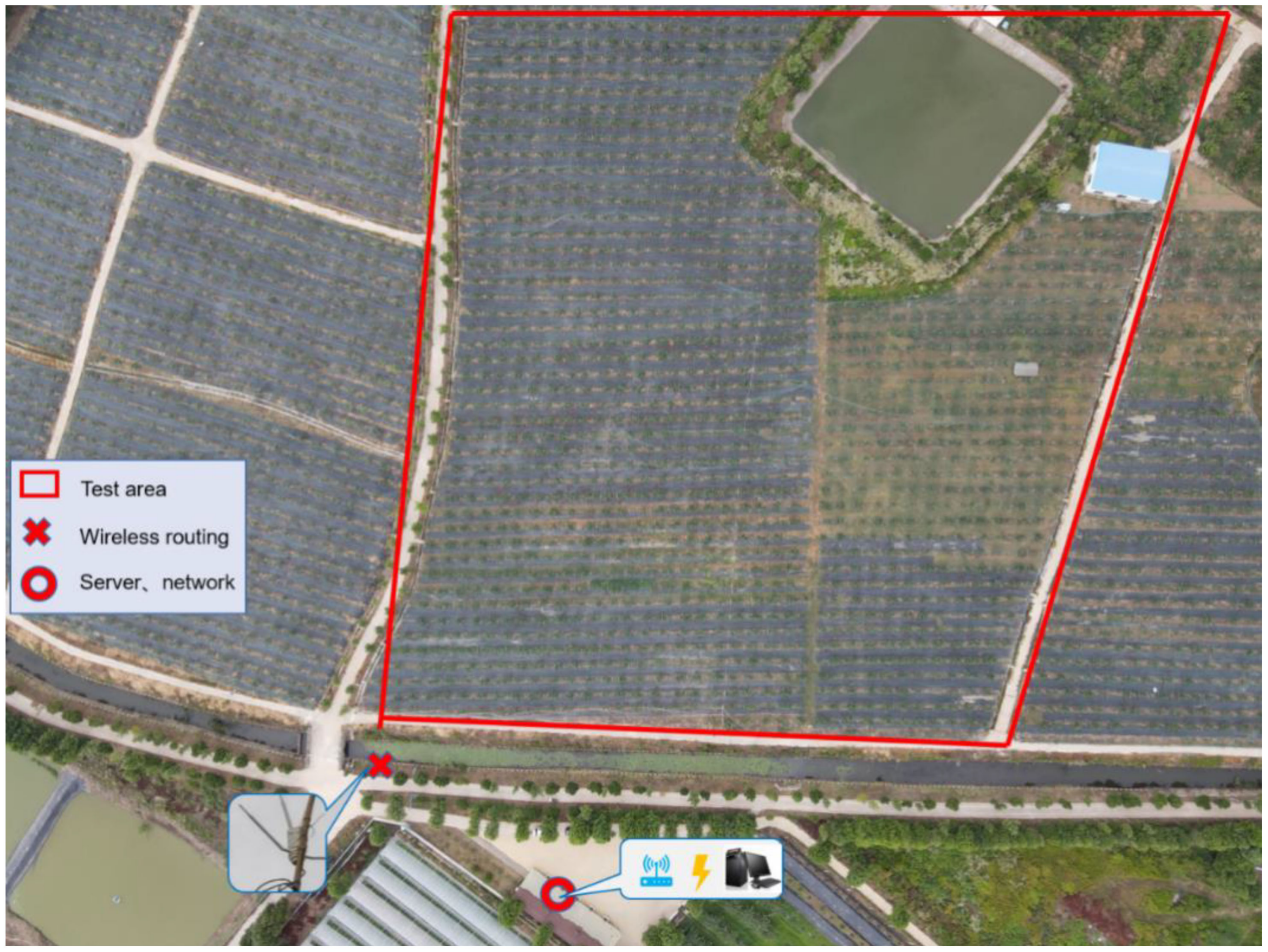
Aerial view of the Pear Garden. The red circle shows one of the houses in the pearly gardens, where power and internet are provided and where the server for the experiment was placed. The quadrilateral box represents the area for this experiment. The location of the crosses is the outdoor router.

The pear trees in the orchard are generally grown in two layers of fruit, the pear trees are spaced 3m apart in rows, 4m apart in plants and 2.4m high. In order not to interfere with the normal operation of the bird repellent, a height of 4m is appropriate. Outdoor Wi-Fi provides a medium for transmitting information between bird repelling equipment, receiving routed signals from office houses conveniently supplied with the network. The router was wired using POE (Power over Ethernet) and arranged to the roadside to cover the orchard trial area (**Figure 10**).

**Figure 11:**
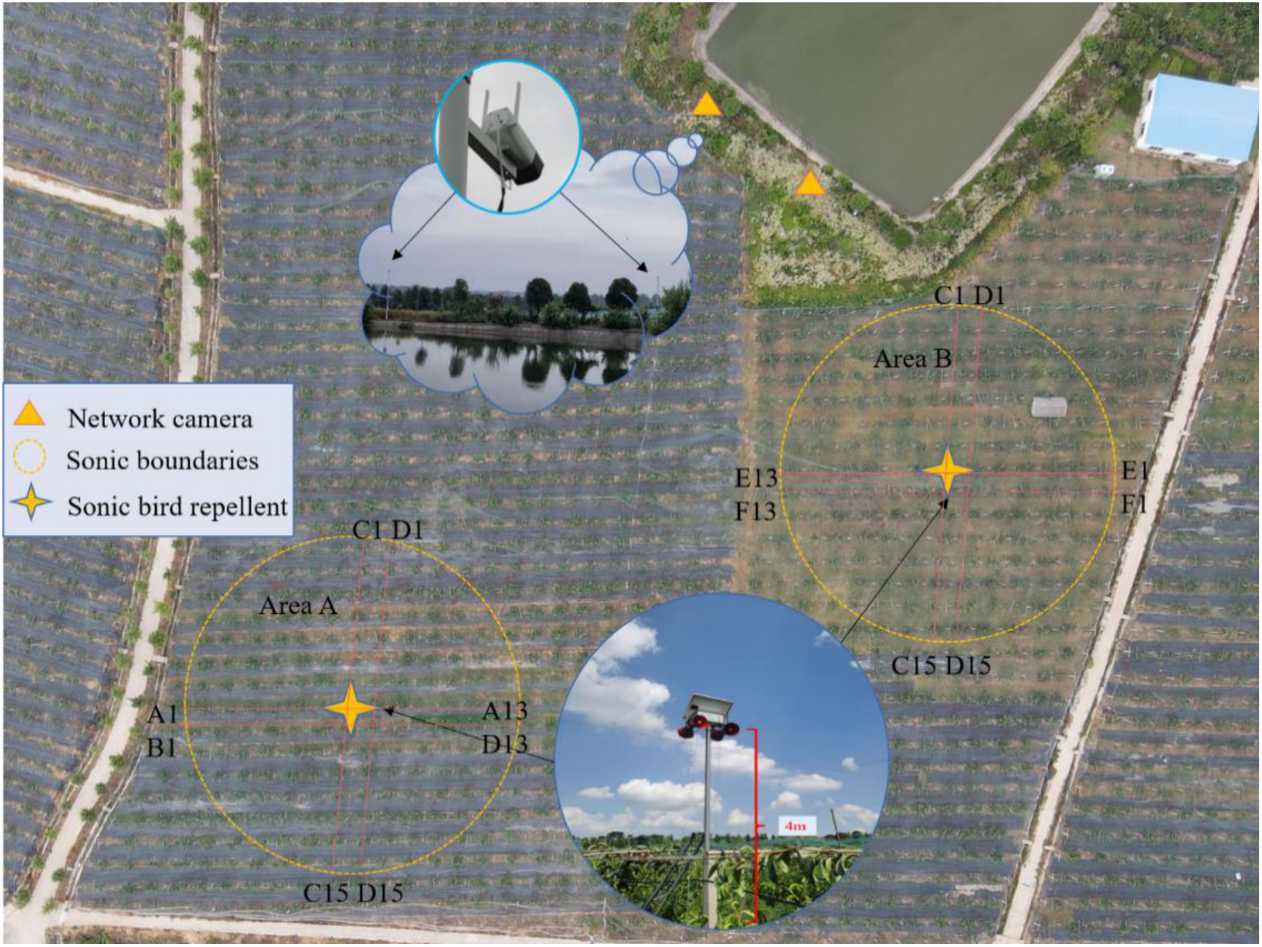
Test arrangement. The yellow circles show the two test areas A and B respectively, with the yellow star pattern in the middle representing where the bird repellent equipment was placed. Two webcams are placed in the triangular area, monitoring each of the two pear orchard areas.

This trial was carried out in the orchard from 13 July to 1 August 2022, dividing the selected pear orchard into two areas as Area A and Area B (**Figure 11**). The external conditions in both areas were essentially the same (Brown and Brown, 2021). To prevent the bird repellent from affecting another area, the two sites were separated by approximately 200 meters in a straight line. Area A was fitted with bird repellents designed in this paper, which automatically recognized birds and triggered the repellents to operate, while area B was fitted with acoustic devices that were manually switched on and off 24 hours a day. Based on previous measurements, the effective radius of the acoustic bird repeller was about 24 meters, while the row spacing of the fruit trees in the pear orchard was 3 meters and the plant spacing was 4 meters. For statistical convenience, the number of fruit trees within the circular coverage area was simplified by taking two columns of 13 and 17 fruit trees horizontally and vertically, respectively, and using marker ties to mark each pear tree. Changes in the number of birds found in the two areas at various times of the day were also counted during the trial, based on webcam footage and field observations.

**Figure 12:**
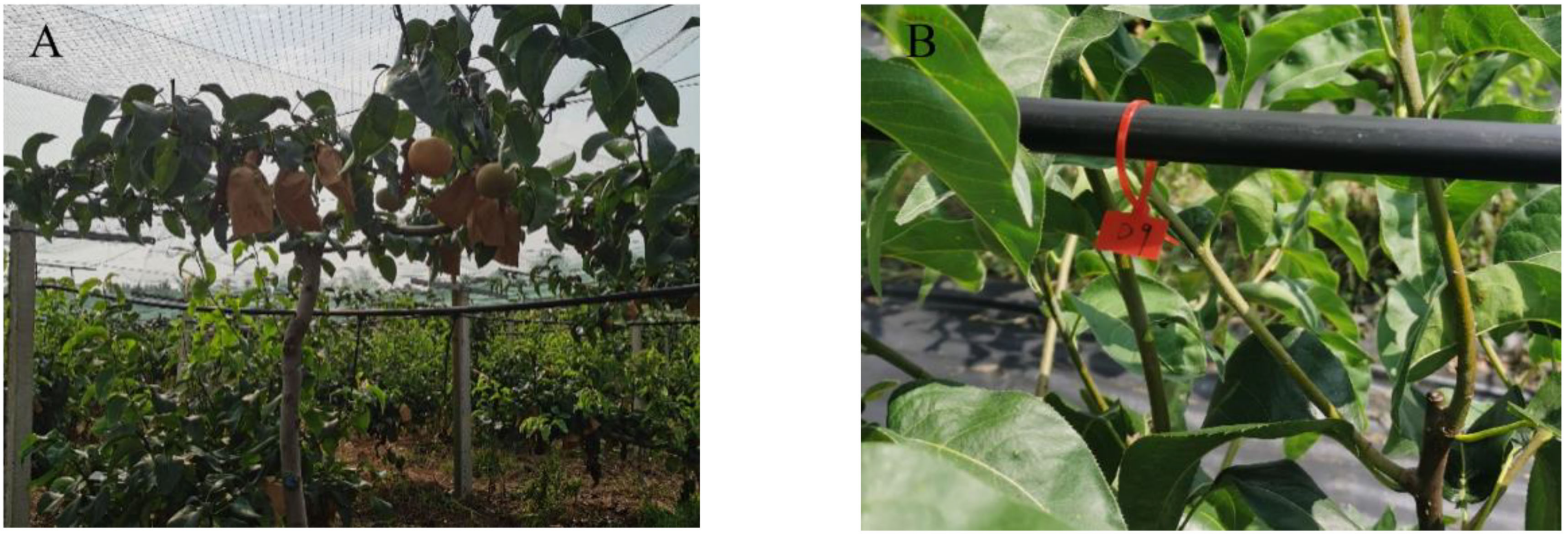
**(A)** Bird netting and fruit bags on pear trees. **(B)** The pear trees in columns A, B, C and D in each area were marked by attaching marker tape to a pipe above each pear tree that provided water and nutrients to each tree.

In addition to being covered with bird-proof nets, the pears on each fruit tree were covered with a kind of paper bag (**Figure 12**). These bags are designed to make the surface of the pears less prone to bruising and to protect them from pests and diseases, which is very helpful in improving the quality of the fruit. However, in order to eliminate the impact of this on the trial and to provide more attractive fruit during the trial period, we removed the protection of the area from the fruit prior to the trial, including bird netting and fruit bags. We recorded separately the number of unbagged fruit on each pear tree and the total number of fruit in each of the two areas. The number of pecked fruit trees in the two groups of zones was counted every two days. In addition to triggering the bird repellers, the webcam can easily record the number of birds passing through the corresponding areas every day. The practicality of the designed bird repellent and the impact of bird adaptation were judged by comparing indicators such as the growth rate of pecked fruits and the ratio of pecked fruits to the total number of fruits in areas A and B.

**Figure 13:**
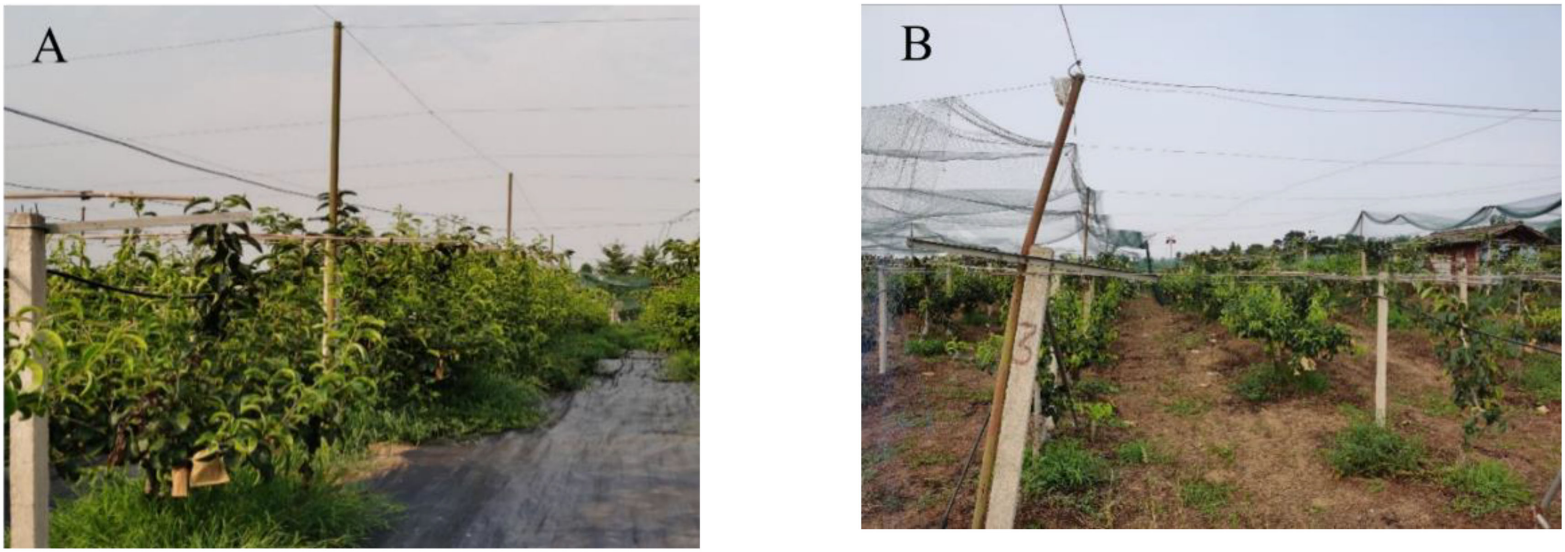
Two areas of pear growing conditions. Area **(A)** was covered with a layer of mulch to stop the growth of weeds, but the roots of the pear trees were high and dense, and the pear trees had dense foliage. Area **(B)** was not covered with mulch, but there were few weeds and the pear trees had sparse foliage.

The number of pears picked from bags in the two areas was 197 and 201 respectively. Through observation, it was found that the planting environment in area A and area B were slightly different, and the pear trees had some different growth conditions (**Figure 13**).

The slight difference in the growth of the pear trees between the two areas may have had some effect on the trial. In order to eliminate the interference of environmental differences in the plots, the test was therefore conducted in two plots where different acoustic bird repellents were tested in rotation. The computer vision based IoT acoustic bird repellent designed in this paper was placed in area A 4 days prior to the trial as planned. An always working acoustic bird repellent is placed in area B. The bird repellents in both areas are then silenced together for 4 days.

## 3 Testing and results

The experiment was conducted for 12 days from July 18 to July 29, with sunrise and sunset around 5:30 and 19:30 BST, respectively, and about 14 hours of sunshine per day. The number of birds present over the two areas was recorded every two hours each day and plotted against the mean value. The total number of birds present each day during the trial was recorded and plotted.

**Figure 14:**
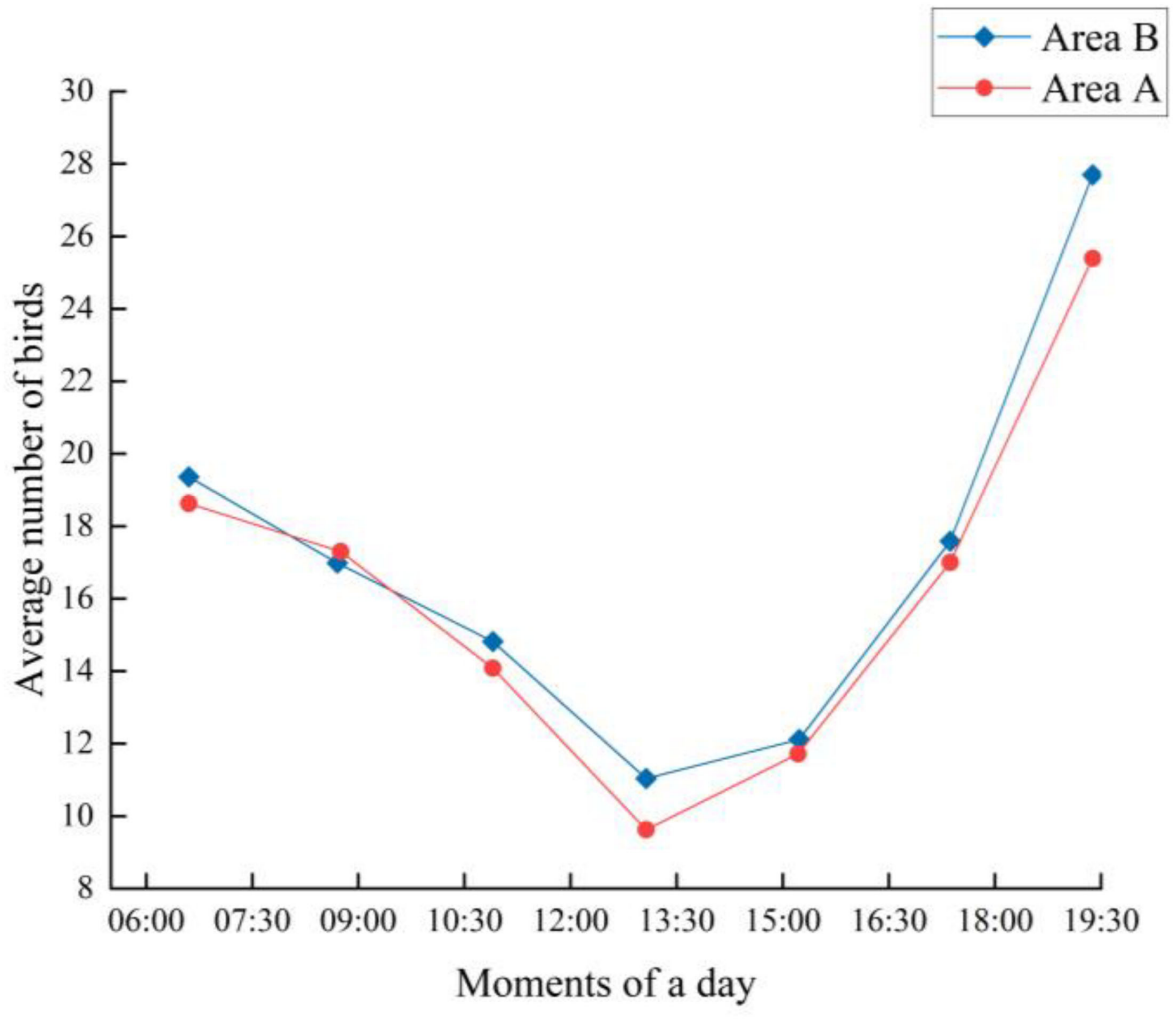
Average number of birds at different times of the day.

As can be seen from **Figure 14**, the average daily frequency of occurrence of birds in Area A and Area B was roughly “V” shaped. Birds appeared more frequently in the morning and evening than in the middle of the day, which reflects the pattern that birds prefer to feed in the morning and evening. The maximum temperature during the test dates was around 42 degrees Celsius and the average temperature was around 31 degrees Celsius, which does not exclude the effect of high midday temperatures on the foraging activity of the birds.

During this period also counted the number of bad fruit on each pear tree pecked by birds from the plucked bags every 2 days (**Table 6**).

**Table 6:** Number of bad fruit pecked by birds in both areas during this trial.

No.	Date	Number of pears pecked					
		Area A			Area B		
		Keep working acoustic bird repellent	None	Ours	Keep working acoustic bird repellent	None	Ours
1	Day1~2			3	8		
2	Day3~4			5	12		
3	Day5~6		11			32	
4	Day7~8		17			60	
5	Day9~10	21					67
6	Day11~12	34					79

It can be seen that 5 new bad fruit were pecked in the first 4 days in area A, while 12 new bad fruit were added in area B. The number of new bad fruit was higher than in area A. In the case of the same influencing factors such as pear tree species and ripening period in both areas, the analysis of the possible reasons are twofold: firstly, the previously mentioned area B is less weedy and sparser than area A, so that the birds can see the fruit on the pear tree more easily and do not block the birds’ observation of their surroundings when feeding to facilitate their escape. Secondly, the acoustic bird repellent that had been working for 4 days had acclimatized the birds in the vicinity.

**Figure 15:**
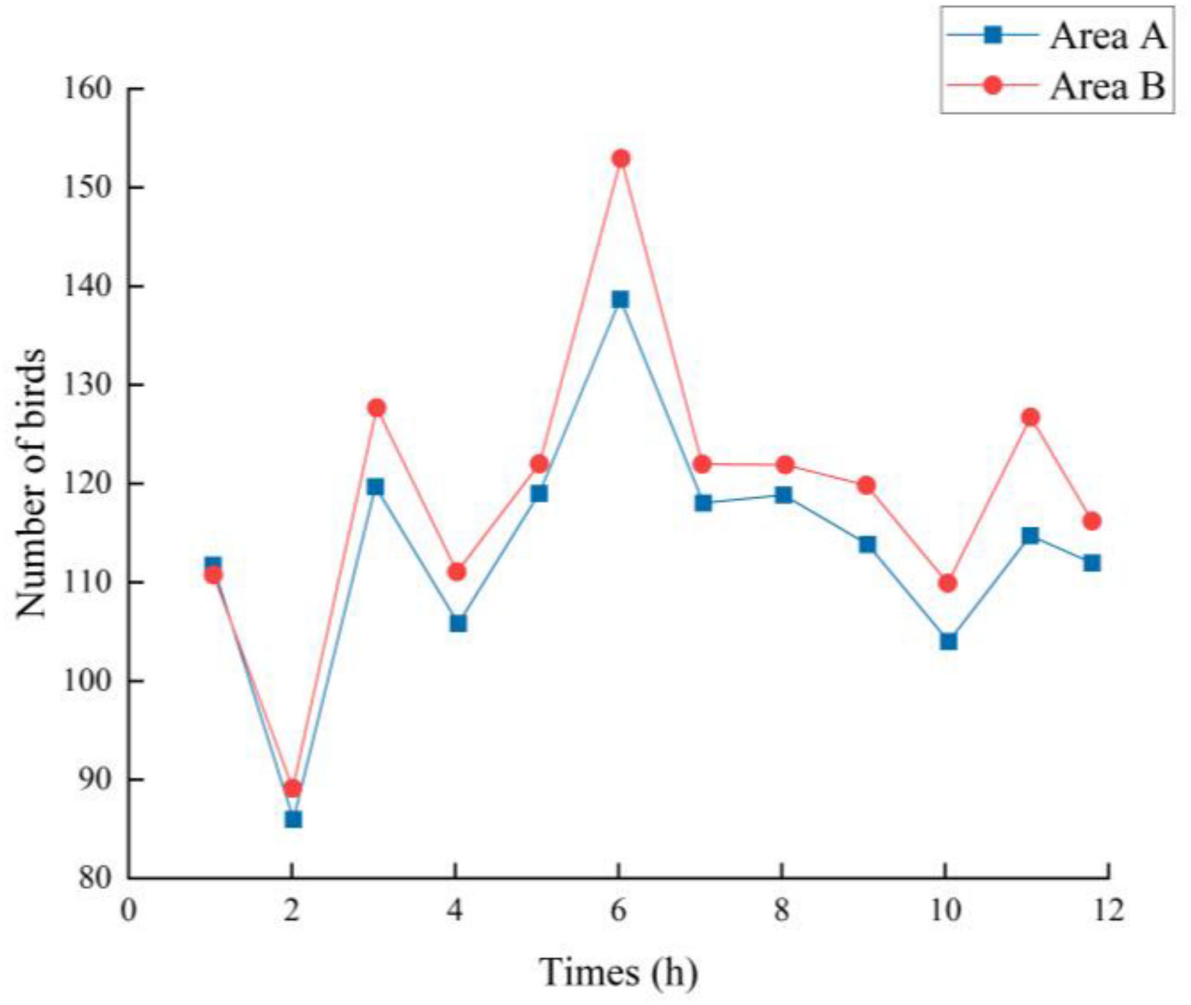
Daily variation in total bird count during the trial.

Between days 5 and 8 of the test period, when bird repellents stopped working in both areas, the number of new bad fruits produced in area B increased to 32 at the first count when the bird repellents stopped working, which was an increase of 20 fruits, or 167%, over the previous count. It can also be seen in **Figure 15** that the total number of birds present during the day reached its highest value on day 6 when bird repellents stopped working in both areas. By the time of the second count, the total number of bad fruits had reached 60. Compared to the period when the acoustic bird repellers were used all the time, there were 48 new bad fruits in the period when the repellers were not used, which was much higher than before the placement of the repellers. Thus, the effectiveness of the universal acoustic bird repeller in suppressing bird pecking can be confirmed. During this period, the 48 new bad fruit in area B were much higher than the 12 new pecked fruit in area A. Two reasons for this can be analyzed: firstly, birds do not easily adapt to the acoustic bird repellent used in area A, which works better based on computer vision triggers. The second is the influence of the environmental factors presumed to be growing in the previous section, with taller weeds blocking the birds’ view of their search for food. Whereas, as can be seen in **Figure 15**, the total number of birds present in area A on day 6 remained high, the second reason for analysis is more likely.

**Figure 16:**
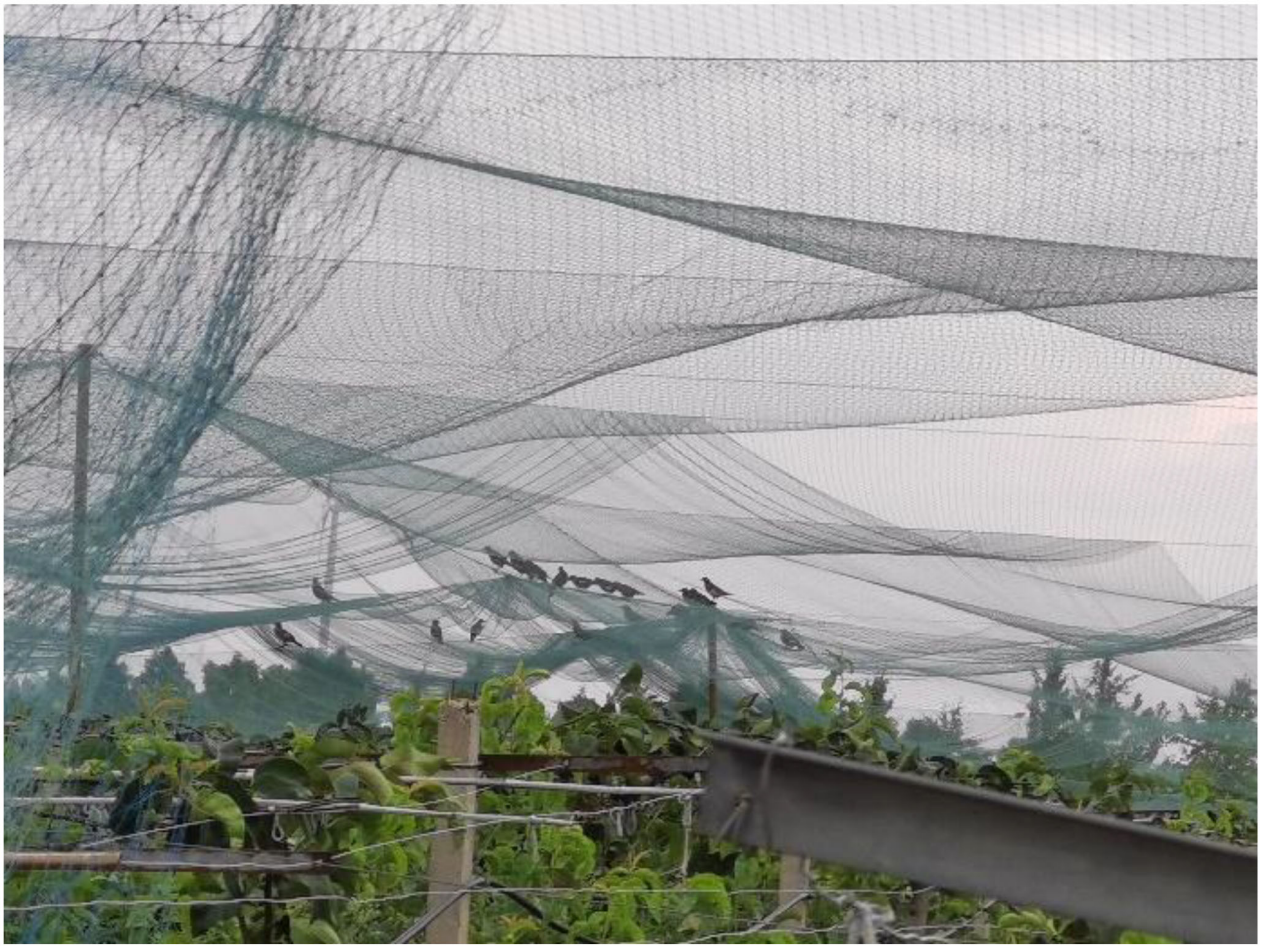
Birds in the vicinity of area A on days 9 to 12.

During days 9 to 12, the devices used in the two areas were switched, with a generic always-working acoustic bird repellent used in area A and a computer vision-based acoustic bird repellent used in area B. During this period, only 7 new bad fruit were added to Area B at the first count, a rate of 11.67%. This increased to 79 pecked fruit at the second count, with a total of 19 new pecked fruit added during this period. Although the number of new pecked fruit was lower than in area B, the rate of new pecked fruit was much higher than in area A, where the acoustic bird repellent was used. In addition to this, there was a higher concentration of birds in Area A (**Figure 16**), which showed no apparent fear despite the fact that the repellents were working all the time.

The use of this split-field trial better excluded the influence of other unrelated influencing factors on the experimental results, the number of pears removed from bags in area A and area B was 197 and 201 respectively, and the total number of fruits removed from bags in the two areas was 398. In a 12-day trial, the total number of pears pecked in both areas during the use of the visually triggered acoustic bird repellent designed here was 24, the total number of pears pecked in both areas with the always working acoustic bird repellent was 29, and the total number of new bad fruit in both areas without the repellent in operation was 52.

**Figure 17:**
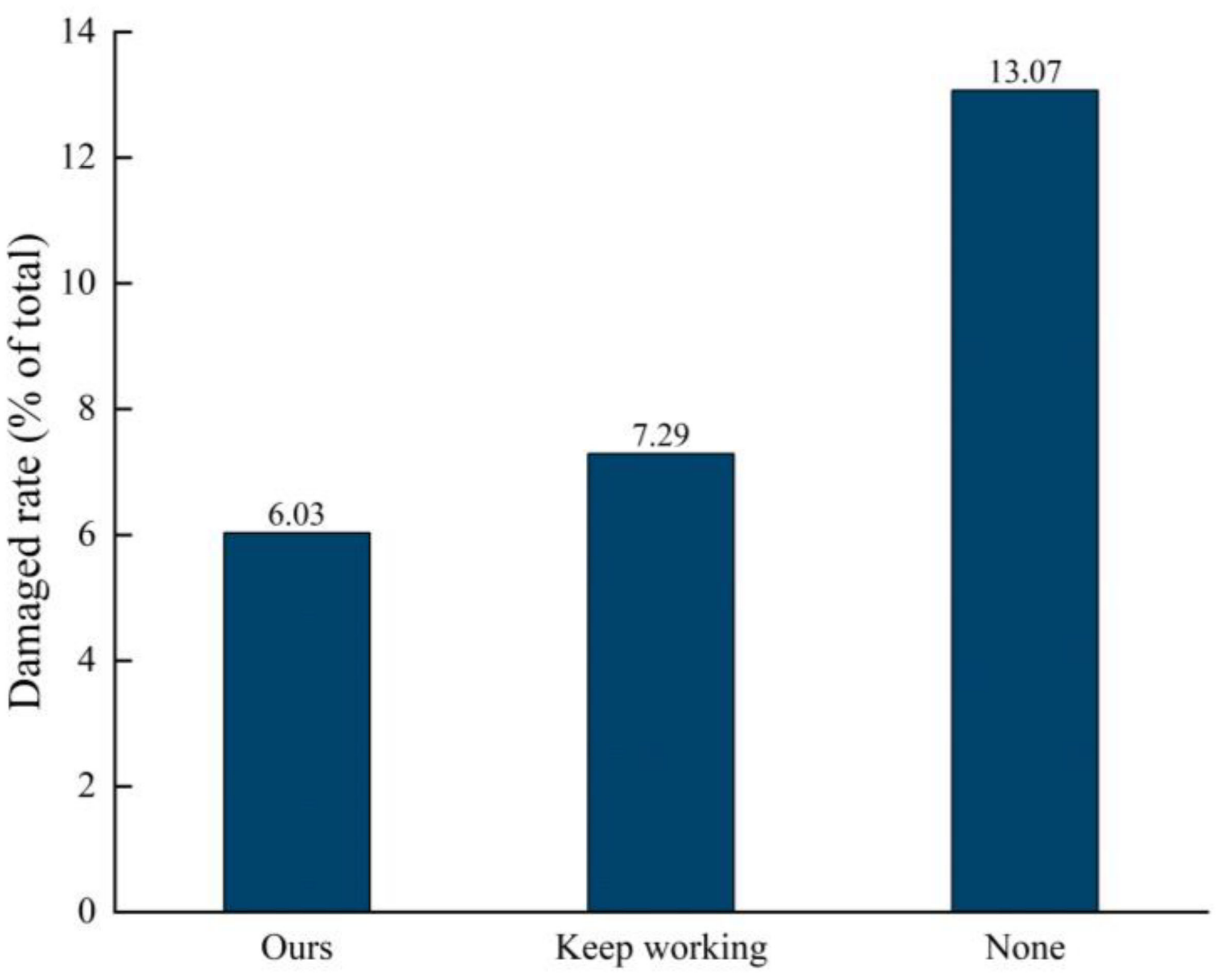
Comparison of bird pecking rates between treatments.

As can be seen in **Figure 17**, there were significant differences in the effectiveness of the different bird repellent programs on bird-infested pear orchards. Using the computer vision-based acoustic bird repeller designed in this paper, 6.03% of pear fruits were damaged during the test period, 7.29% of fruits were damaged during the test period using the continuously working acoustic bird repeller, while the damage rate of pear fruits without using any bird repellent tool was 13.07%. The data shows that both the acoustic bird repellent designed in this paper and the acoustic bird repellent that has been working have some effect in repelling birds. Using the computer vision-based acoustic bird repellent designed in this paper resulted in a 53.86% reduction in bird pecking rates compared to the blank group, and 44.22% reduction in bird pecking rates compared to the blank group using the acoustic bird repellent that worked non-stop all the time.

**Figure 18:**
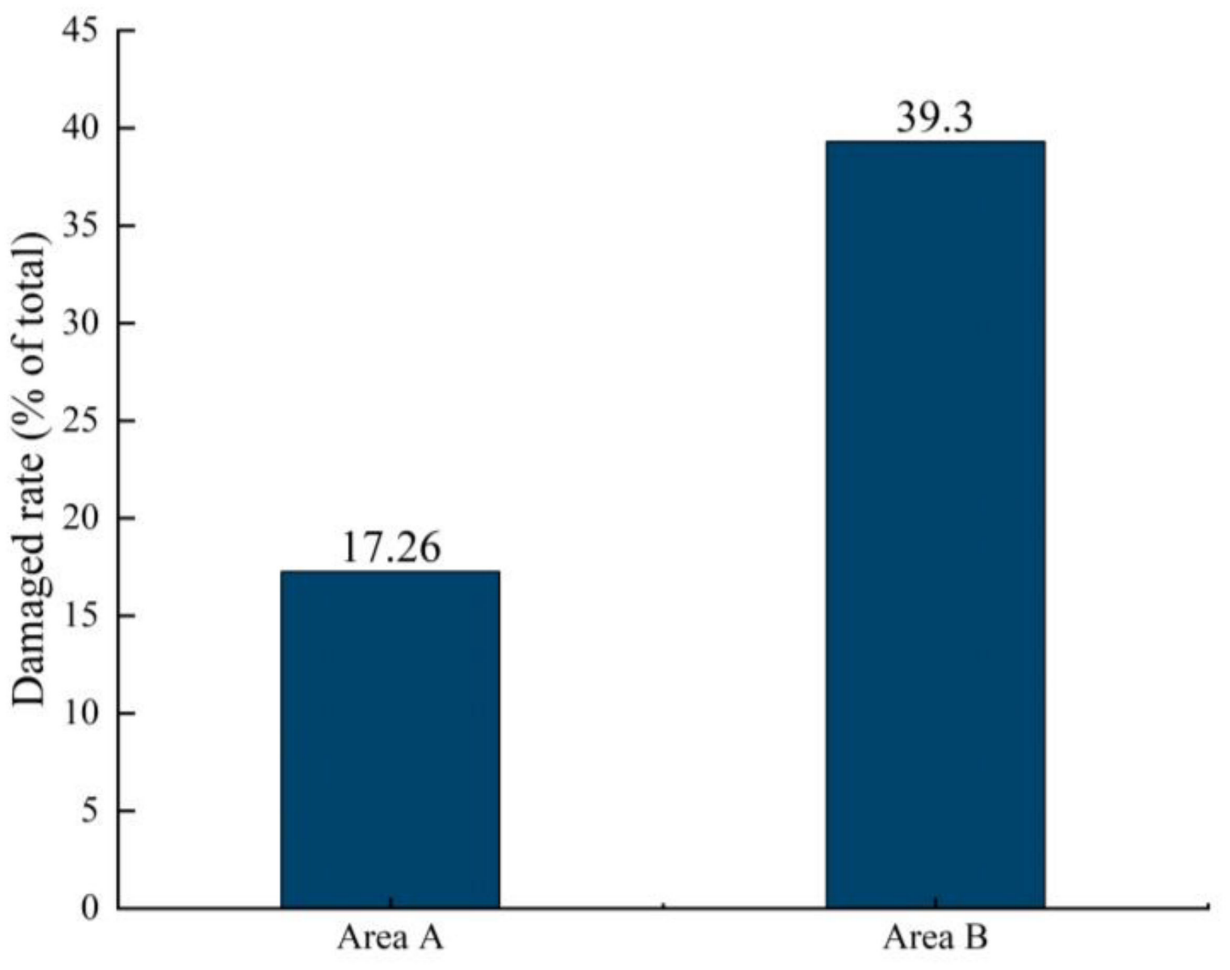
Regional domain A in relation to pecking rate.

At the last count of fruit pecking in **Table 6**, 34 pears were pecked in area A and 79 in area B. The pecking rates were 17.26% and 39.30% (**Figure 18**), respectively. Pears in area B were more susceptible to bird infestation, with 56.96% less fruit being pecked in area A than in area B. In addition to the differences in the treatment order of the two areas of the test program, the biggest influencing factor is the different pear growing environment.

## 4 Discussion

The ultrasonic bird repeller designed in this paper is triggered by computer vision and has high recognition accuracy. As soon as an invasive bird is detected in a pear orchard, the device starts working and emits sound waves that are unbearable for birds to repel them away. Birds are very good at adapting to human stimulation of their senses such as smell, hearing and sight, but require a long exposure time to adapt. The device designed in this paper is unique in that it works intermittently. In this test conducted in a pear orchard, the damage rate of pears using the device was 6.03% during the test period, whereas the damage rate of pears with the acoustic bird repeller working uninterruptedly was 7.29% during the test period, and the damage rate of pears without the use of any bird repellent tool was 13.07%. It is clear that the acoustic bird repellers are very effective in reducing the infestation of pear orchards by wild birds.

It was found that the acoustic bird repellers in the control group had 61.90% new bad fruits at the last count, which was much higher than the 25.53% new bad fruits of the acoustic bird repellers which used computer vision during the same period. In addition, despite the fact that the bird repellers were working and sounding alarms all the time, significant aggregations of birds were observed in the neighborhood. This is similar to the findings of Bomford (1990), but it is not sufficient to suggest that the sound of the acoustic bird repellent was completely ineffective against the birds, as the first few days of the trial did not show this gathering of birds. Despite the different high-decibel audio emitted by acoustic bird repellents, birds appear to adapt more readily to the environment under such sound waves due to the length of time they are in contact with the sound waves emitted by uninterruptedly operating acoustic bird repellents, which can lead to repellent failure (Howarth, 1991). The use of acoustic bird repellent should minimize the time between birds and acoustic stimuli and be unpredictable, which may reduce adaptation to some extent.

**Figure 19:**
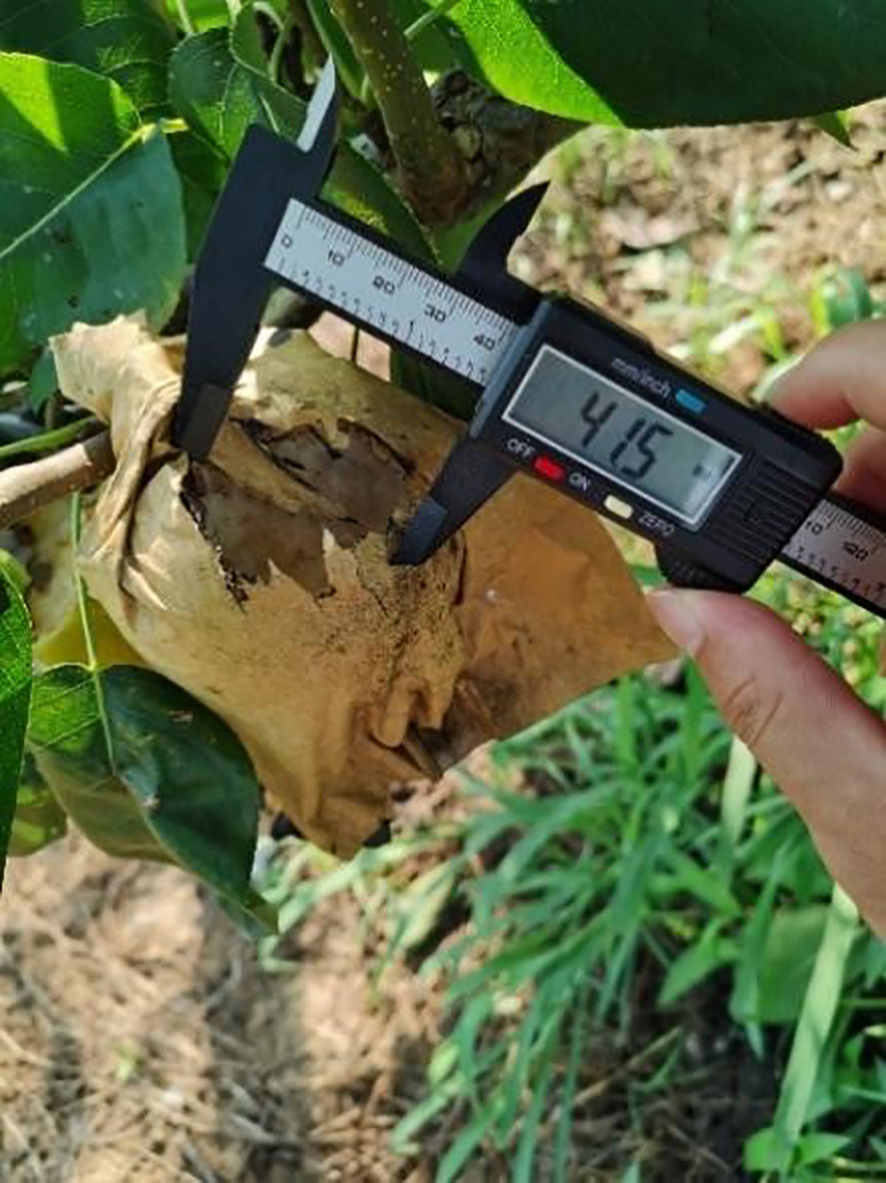
Only a few of the bagged pears were pecked.

The growth of pear numbers (**Figure 9**) can be divided into upper and lower layers, and when counting the number of pears pecked by birds during the trial, we found that the number of bad fruits in the upper layer of the pear tree was significantly higher than the number in the lower layer. Birds that hover in the sky to feed were more likely to spot growing fruit from above, and they were also more likely to be observant of their surroundings as well as alert to the approach of predators because of their wide field of vision when feeding from high above. In preparation for the trial, we removed a certain number of bags of fruit from the pear trees in both areas. At the final count, only a few bagged fruits were pecked (**Figure 19**) by the birds and most of the fruits were pecked with the bags removed. And in the split-field experiment, the pear trees in area A had higher root weeds and denser foliage than in area B, making the fruit less likely to be found (Avery, 1989). The pear pecking rate in area A and area B was 17.26% and 39.30% respectively, pears in area B were more susceptible to birds and the fruit in area A were 56.96% less susceptible to pecking than those in area B. Birds seem to prefer food that is exposed and easy to see. As fruit shading conditions were not part of the study for this trial, additional studies will be required in the next study to determine the certainty of this assessment.

Although the study design in this paper was sound, the trial used only one orchard, which limits the generalizability of this study in terms of geography, season, and fruit type (Elbers and Gonzales, 2021). More research could be conducted in the future to optimize bird repellents and extend bird acclimation, and some possible directions for research are listed below:

1. Optimize the design aspects of the bird repeller. The bird repellent system can be improved by further exploring and improving the technical aspects of human-computer interaction interface, ultrasonic voice and cooperative work. For example, to enhance the orchard multi-point synergy of the bird repellent system, and to construct the orchard multi-point synergistic bird repellent system.
2. Extend the adaptability of birds to bird repellents. Based on the ease of adapting to a single bird repellent, multiple bird repellents are integrated, such as light bird repellents, motion sensors, and the addition of bird repellent chemicals. Since birds are sometimes adapted to specific frequency sounds or sound waves, the frequency and pattern of sound waves emitted by bird repellent devices can be altered to prolong the birds’ adaptation to bird repellents. In addition to this, the location of the bird repellers can be changed periodically, etc.

## 5 Conclusion

In this paper, an acoustic bird repellent triggered by computer vision was designed and tested in a pear orchard in a separate field. The findings suggest that birds are prone to adaptation when exposed to acoustic stimuli for longer periods of time. Compared with the blank group, the acoustic bird repeller based on computer vision designed in this paper reduced bird pecking by 53.86% and 44.22%, respectively, and it is clear that the acoustic bird repeller is very effective in reducing the invasion of wild birds into the pear orchard. Computer vision-triggered ultrasonic bird repellents work intermittently, and the use of computer recognition-based acoustic bird repellents is less likely to be adapted by birds than acoustic bird repellents that work all the time. In addition, in order to minimize the economic losses caused by bird damage, fruit bagging can be used together with sonic bird repellers for better bird repellent results.

## Data Availability

The raw data supporting the conclusions of this article will be made available by the authors, without undue reservation.

## Supplementary material

Supplementary Figure S1 Birds entangled in bird-proof nets.
