# Supplementary figures and images for: An experimental study of acoustic bird repellents for reducing bird encroachment in pear orchards
Source: Front Plant Sci. 2024 Sep 9;15:1365275. doi: 10.3389/fpls.2024.1365275 (PMC11416946; doi:10.3389/fpls.2024.1365275)

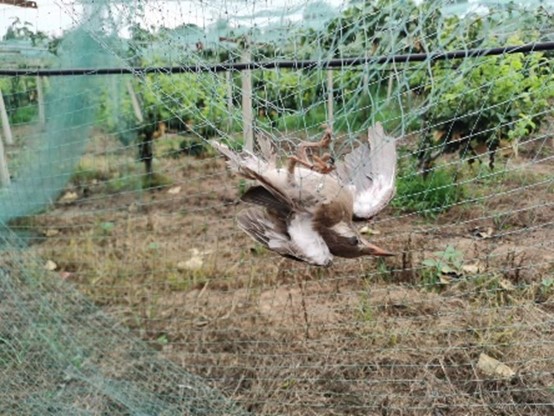

Supplement: Supplementary Figure S1 — Birds entangled in bird-proof nets. [file Image1.jpg]
