# Supplementary material for: An experimental study of acoustic bird repellents for reducing bird encroachment in pear orchards
Source: Front Plant Sci. 2024 Sep 9;15:1365275. doi: 10.3389/fpls.2024.1365275 (PMC11416946; doi:10.3389/fpls.2024.1365275)
Supplement: Supplementary file 2 [file Table1.docx]

| **Table S1**  Webcam Parameter List | | |
| --- | --- | --- |
| Webcam Picture | Parameter | Value |
| 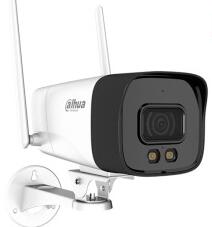 | Resolution | 1080P/2K |
|  | Networking Method | Wi-Fi/WLAN |
|  | Focal length | 6mm |
|  | Storage Method | Memory Card / Cloud Storage / Hard Disk |
|  | Waterproof rating | IP67 |
